# Metamorphic Evidence as a Runtime Reliability Signal for Small Language Models

**DOI:** 10.3390/s26216918

**Published:** 2026-10-31

**Authors:** Bongjin Jung, Joongho Chang

**Affiliations:** 1 Program in AI Convergence Engineering, aSSIST University, Seoul 03767, Republic of Korea; eugene_jung@stud.assist.ac.kr; 2 Program in AI Convergence Engineering, SDG Management School, 1202 Geneva, Switzerland

**Keywords:** black-box monitoring, small language model, selective prediction, metamorphic testing, edge inference, label-free confidence, quantized inference, mathematical reasoning

## Abstract

Small language models often run locally, where no larger model can check their answers, and the runtime exposes tokens and log-probabilities but not hidden states. MOME (Module of Metamorphic Evidence) is a label-free runtime gate. It edits each problem twice by deterministic rules, once keeping the correct answer and once changing a condition on which that answer depends. A predictor fitted offline on other backbones scores the answers. A two-stage policy serves high-scoring answers and verifies the rest by a vote over further samples or declines them. Mathematical word problems were used because every answer can be checked exactly. On five four-bit backbones and three benchmarks, the gate raised selective accuracy from 69.5% to 85.8% with two stage-2 samples and to 88.1% with four, at 4.34 and 5.70 generations per item. Correct yield stayed at the ungated level. The MOME score ranked answers with an area under the ROC curve (AUROC) of 0.863, against 0.734 for mean token log-probability. On four backbones of further families, it reached 0.829 against 0.629, and correct yield changed by −1.81 percentage points at stage-2 temperature 0.7 and by −0.19 at 0.3. The fitted components apply to mathematical problems, but the procedure can be reused in other domains.

## 1. Introduction

### 1.1. Deployment Setting and Detection Question

A growing share of language-model inference runs outside the data center, close to where the request is made [1,2]. Such hosts include edge servers and gateways that process requests near the devices and sensors that produce them. Such environments have limited memory and compute, so they use small models whose weights are quantized to low bit widths [3,4]. In this paper, a *small language model* is a backbone that can be served locally with four-bit weights. The evaluated backbones have 1.5 to 11 billion parameters. The runtime of this study returns the generated tokens and their log-probabilities but not the weights or the hidden states, so classifiers trained on internal activations cannot be applied. This study concerns what the runtime makes observable, not the hardware.

The study also assumes that no larger model can check or replace an answer. Examples are an offline host and a setting where cost or data protection rules out a hosted model. The system can then answer an item, decline it, or query the same model again. Additional generations are the only extra resource, so cost is measured in *generations per item*. This paper asks how such a system, with no ground-truth label, grader, hidden states, or larger model, can detect that an answer is wrong. It also asks whether a gate built on this detection can raise the accuracy of served answers without lowering the number of correct answers, and at what cost in generations.

### 1.2. Mathematical Problems as a Controlled Test

A detector of wrong answers can be tested only if the correctness of every answer is known. The study was therefore restricted to mathematical word problems, in which an answer is correct only if it equals one definite reference answer. The error rates of the backbone and of the detector could then be measured exactly, without a judgment by a person or a second model. Arithmetic also fixes in advance how an edit affects the correct answer so that the edits could be written as deterministic rules.

For the same reason, the rules, the fitted score, the thresholds and the measured gains apply only to mathematical word problems. They do not transfer directly to tasks whose answers are open or must be judged. A procedure of four steps can transfer.

1. Write edits whose effect on the correct answer is known before the model answers.
2. Record for each item the answers that stayed the same and those that changed (the fingerprint of the item).
3. Fit a score on the fingerprints of backbones other than the evaluated one.
4. Judge the resulting gate by correct yield under an adoption rule registered in advance.

Section 5.3 states what a study in another domain would have to supply.

### 1.3. Output Statistics in This Evaluation

In this evaluation, the output statistics exposed by the runtime separated correct from wrong answers above chance. Their separation was weaker than that of the score of the proposed Module of Metamorphic Evidence (MOME; Section 3.3). Five statistics were computed from the generated tokens and their log-probabilities. For each statistic, the area under the receiver operating characteristic curve (AUROC) measured how well it separated correct from wrong greedy answers. All 4497 evaluated items were used, in 15 cells of five backbones and three benchmarks (Section 3.6). An AUROC of 0.5 means no discrimination.

The AUROC was 0.734 (95% interval 0.719–0.749) for mean token log-probability, 0.683 (0.668–0.698) for minimum token log-probability and 0.667 (0.651–0.684) for answer-span log-probability. The answer span is the set of tokens of the extracted final answer, and its statistics are means over these tokens. Entropy is computed over the ten most likely tokens at each position. Entropy and length measure uncertainty, so they were ranked in reverse, which gave 0.668 (0.651–0.685) for answer-span entropy and 0.575 (0.556–0.593) for generated length. The MOME score reached 0.863 (0.850–0.876) on the same items. Its item-paired difference from each statistic was between 0.128 and 0.288, and the 95% intervals of all five differences excluded zero. Within each backbone, the best output statistic reached between 0.748 and 0.871, and the MOME score was higher on all five backbones (Figure 1).

On the 1415 answers that stage 1 of the gate served immediately, the five AUROCs were between 0.51 and 0.59. These answers were selected by a cutoff on the MOME score, which contains mean token log-probability as one of its features. The range of the signals on this subset was therefore restricted, and the MOME score itself reached only 0.467 there. Every comparison in this paper therefore uses the values on all items.

The five statistics are confidence proxies, and the runtime returns no other information about the model’s uncertainty. This result does not mean that the model produces no signal of correctness. It means only that, in this evaluation, these statistics ranked answers less well than the MOME score. Prior work has studied the calibration of model confidence under distribution shift [5], in question answering [6] and in verbalized form [7], and has found modern neural networks to be poorly calibrated [8]. These studies concern calibration, the agreement between confidence and observed accuracy. The measurement in this study concerns discrimination, the ranking of correct above wrong answers.

### 1.4. Metamorphic Relations as a Runtime Signal

A deployed system without labels cannot compare an answer with the correct answer. For some edits, however, the effect on the correct answer is known in advance: the answer must stay the same or *must change*. Metamorphic testing asserts such relations between outputs instead of checking outputs against ground truth [9,10]. This approach addresses the oracle problem, the lack of a reliable way to decide whether an output is correct. This study uses two families of metamorphic relations, one for each direction of change.

- must-hold:an edit that keeps the meaning of the problem, so the correct answer must stay the same. In this study, the edit appends a redundant clause stating that all quantities are exactly as given. This edit is also called the *invariant edit*.
- must-change: an edit that alters a condition on which the answer depends. Examples are a reversed comparison, a scaled quantity, and an exchanged extremum. If the rule applies as intended, the correct answer changes. Section 3.2 states when the correct answer may not change.

A generation of the backbone on an edited problem is a *probe*, and the answers to the two probes are called the *metamorphic fingerprint* of the item. Table 1 shows both edits on one problem. Invariant edits alone miss one failure, in which the backbone ignores a changed condition and reproduces a memorized result. Such a backbone gives the same answer under an invariant edit, and only a must-change edit can detect the failure. Section 4.10 reports how much this check contributed in this evaluation.

### 1.5. From Score to Decision

MOME (*Module of Metamorphic Evidence*) converts the fingerprint into a decision for each item and does not ask the backbone to state its confidence (Figure 2). Stage 1 generates the greedy answer and the probes, and serves the answer if the MOME score of the fingerprint is high enough. Otherwise, stage 2 draws *k* self-consistency samples [11], two or four in this evaluation, and serves the majority answer of the k+1 votes or declines the item. Stage 2 therefore adds cost only for the items that stage 1 does not decide.

### 1.6. Contributions

1. Two families of rules, for invariant and for must-change edits, defined by the arithmetic of word problems. The MOME score is computed from the resulting fingerprint without a label at inference time (Section 3.2 and Section 3.3).
2. A deployable two-stage policy (serve, verify, decline) whose cost per item is a fixed part plus a term that depends on how consistently the backbone answers. In this evaluation, the cost was between 3.69 and 4.98 generations per item (Section 3.4 and Section 4.3).
3. An evaluation protocol registered before the evaluation runs, with *correct yield* as the success criterion, so that a gate cannot succeed by declining more items (Section 3.1 and Section 3.5).
4. A comparison of the MOME score with a linear probe on hidden states obtained outside the runtime (Section 4.12 and Section 5.2).
5. A procedure for testing metamorphic evidence in other domains, which separates the parts that depend on arithmetic from the parts that do not (Section 5.3).

Metamorphic relations, selective prediction, and self-consistency have each been studied (Section 2). To the authors’ knowledge, they have not been combined as in MOME for a deployment without hidden states and without a larger model. MOME generates edits in both directions by rule and uses the answers at runtime as a per-item fingerprint, instead of aggregating them offline over a benchmark. In this evaluation, the invariant edit carried most of the score, and the must-change edit added little (Section 4.10). It feeds the fingerprint to a two-stage policy, which was judged against a preservation criterion for correct yield registered in advance.

## 2. Related Work

### 2.1. Selective Prediction and the Reject Option

The trade-off between error and abstention was analyzed by Chow [12] and has a learning-theoretic basis [13]. Deep selective classifiers threshold a confidence score [14] or integrate the reject option into the network [15], and the maximum softmax probability serves as a baseline confidence score [16]. Conformal risk control bounds the expectation of a monotone loss [17]. Conformal language modeling calibrates a stopping rule so that the returned set contains an acceptable answer with a chosen probability [18]. These distribution-free guarantees require a labeled calibration set drawn from the deployment distribution. The thresholds of MOME were also fitted offline on labeled items, but backbone-out (Section 3.3), so they carry no coverage guarantee. The registered adoption rule of this study instead requires that the gate not reduce correct yield.

### 2.2. Metamorphic and Behavioral Testing

In natural language processing, metamorphic relations serve as *evaluation* frameworks that test the behavior of models [19] and, when grounded in logic, the reliability of their reasoning [20]. The closest prior work, MarMot, turns domain-specific metamorphic relations into an online monitor for autonomous driving and interprets relation violations as runtime uncertainty [21]. MOME applies this approach to language models with two changes. The relations of MOME are generated by rules from the input text instead of a physical domain model. MOME also chooses for each item to serve, verify, or decline instead of raising an alarm.

### 2.3. Perturbed Mathematical Reasoning

On GSM-Symbolic, model accuracy decreased when numerical values were changed and decreased more when irrelevant clauses were added [22]. Automated frameworks generate adversarial variants of problems for each target model through iterative rewriting and verification [23]. These works use perturbed problems to build *benchmarks*. MOME uses the same class of edits at deployment as a signal that an individual answer may be wrong. The edits are produced by deterministic rules instead of by another model (Section 3.2).

### 2.4. Self-Consistency and Its Cost

Chain-of-thought prompting improves accuracy on reasoning tasks [24], and sampling several chains and taking a majority vote improves it further [11]. The cost of the vote grows linearly with the number of samples. MOME draws samples only for the items that stage 1 does not serve. In this evaluation, stage 1 served 31.5% of the items without a stage-2 sample.

### 2.5. Label-Free Uncertainty

Consistency across sampled generations detects hallucination without reference texts [25]. Semantic clustering groups the sampled answers by meaning, so that agreement is measured between meanings instead of between strings [26,27]. These methods keep the problem fixed and vary the *sampling*. MOME varies the *problem* with an edit whose effect on the answer is known in advance, which makes must-change edits possible.

### 2.6. Hidden-State Interpretability

In transformer language models, feed-forward layers act as key–value memories [28] and promote concepts in vocabulary space [29], and factual associations can be located and edited [30]. Section 4.12 uses a linear probe on hidden states as an offline comparator for the MOME score, because the deployment runtime of this study gives no access to hidden states.

## 3. Materials and Methods

### 3.1. Problem Setting and Metrics

For each item *x*, the policy takes one of three actions: *serve*, *verify*, or *decline*, the term used here for abstention. Verify is an intermediate action that ends in serve or decline. The *ungated model* is the same backbone, serving its greedy answer to every item. Four metrics are reported.

- **Coverage.** The number of served items divided by the number of all items.
- **Selective accuracy.** The number of correct served answers divided by the number of served items.
- **Correct yield.** The number of items that are correct *and* served, divided by the number of *all* items.
- **Generations per item.** The number of calls to the backbone, probes included, averaged over all items.

*Serve-band precision* is selective accuracy restricted to the items that stage 1 serves immediately (Section 3.4). *Reliability* is the probability that a served answer is correct under the benchmark’s answer-equivalence protocol. Reliability does not imply semantic safety, factual completeness, or robustness beyond the evaluated tasks.

Selective accuracy alone is not a suitable success criterion, because a gate can raise it by declining more items, whereas correct yield cannot be raised in this way. A gate was counted as successful only if its correct yield was at least that of the ungated model. This condition is called *capability preservation*, and it makes no claim about response quality, helpfulness, or transfer to other tasks. The condition is part of the adoption rule that was registered in advance (Section 3.5).

Detection and policy are treated separately, because a result on one does not determine the other. *Detection* is how well a score ranks answers by whether they are wrong. AUROC and the risk–coverage curve (Section 4.2) measure detection without a policy. *Policy* is what the system returns for each item and at what cost. The four metrics measure the policy. The matched-coverage comparison in Section 4.1 separates the quality of the score from that of the gate. Differences between two rates are reported in percentage points, written *p* in the text and *pp* in table headers.

### 3.2. Metamorphic Relations

A rule produces an edit δ that maps a problem *x* to an edited problem $\tilde{x}=\delta \left(x\right)$. Each edit carries the expected response of the answer, E(δ)∈{MUST-HOLD,MUST-CHANGE}, and, where the rule can determine them, its direction and multiplier. The perturbation engine calls no model, so MOME runs at deployment without a second model. The engine applies regular-expression rules in a fixed order and contains no random step, so the same problem text always yields the same edits. The only random element of the method is the stage-2 sampling, which uses fixed seeds. Appendix A traces the engine on two problems.

Three rule packs cover the problem texts of the benchmarks. The English pack contains quantity scaling, comparison reversal, and extremum exchange, and the Korean pack applies the same relations to Korean comparatives and numerals. The LaTeX pack, which runs on every item in addition to the pack for its language, reverses inequality signs and exchanges \max and \min. Table 2 gives example edits from each pack.

Each rule type carries a *rule certainty*, high or medium, which annotates its expected response and is not verified for each edited problem. All reversal rules are annotated *high*. They reverse a comparison, an extremum, an inequality, a direction of change, a sign, a parity, or a factor of two. Examples of the last four are *increased* and *decreased*, *positive* and *negative*, *even* and *odd*, and *twice* and *half*. In a well-formed problem, such a reversal normally changes the correct answer, but it can also leave no valid value or no unique answer.

Doubling a quantity, written in digits or in words, is annotated *medium*, because it changes the correct answer only if the quantity enters the computation. The rule does not check this condition and does not assert that the answer doubles. Agreement in magnitude with the annotated multiplier is therefore recorded as a separate bit of the fingerprint and never counted as a violation. An unchanged answer on a must-change edit of either certainty is counted as a violation.

A *well-posedness* filter restricts where the rules apply. A number written in digits is scaled only if it occurs once, lies between 2 and 100,000, and is not a year between 1900 and 2100. Its doubled value must also be absent from the problem. A number written in words is doubled only if it occurs once and its doubled form is absent. A word pattern is reversed only if it occurs exactly once and only in unambiguous forms. For example, *even* and *odd* are reversed only before *number*, *integer* or *digit*. The LaTeX pack edits the whole text of a problem that contains a LaTeX command. In other problems, it edits only expressions in dollar signs, so that a dollar amount in prose is not edited. The filter does not solve the edited problem. Table A1 reports, for each benchmark, the number of problems for which the filter admits at least one edit.

### 3.3. The Metamorphic Fingerprint and the MOME Score

For each probe, the system records symbolic observations that need no correctness label. They include whether the normalized answer to the edited problem differs from the original answer. For a must-change probe, they also include whether the change agrees with the annotated direction and whether its size matches an annotated linear scaling. The fingerprint is built around two kinds of *violation*. The first is a must-change probe whose answer does not change, which occurs, for example, when the backbone ignores the altered condition. The second is an invariant probe whose answer changes.

A logistic model with $L_{2}$ regularization maps the fingerprint, encoded as 26 standardized features, to the MOME score $c_{\mathrm{safe}}$. The first group of features records, for each kind of probe, whether the answer changed, whether a violation occurred, and whether a change agreed with the annotated direction. The second group records the number of probes, the number of unanswered probes, and truncation. The third group describes the generations: the length ratio and text similarity between the original answer and the probes, and the length and mean token log-probability of the original answer.

The term *label-free* applies to the gate at deployment. At deployment, the gate uses no gold answer, calls no grader, and computes the MOME score only from the outputs of the backbone. The coefficients and the standardization statistics were fitted offline on correctness labels, *backbone-out*. The predictor applied to a backbone was fitted on the items of the other four backbones. No item of the evaluated backbone was therefore used to fit the predictor that scored it. The thresholds of Section 3.4 were chosen on the raw scores of the same predictors, and all results use these raw scores. The released predictor (Supplementary Materials) adds a Platt calibration layer [8,31], which changes the score scale and was not evaluated here.

### 3.4. The Two-Stage Gate

Figure 2 shows the policy and the measured flow of items through it. Stage 1 runs one greedy generation, one invariant probe, and at most one must-change probe on every item. The must-change edit is the first high-certainty edit in a fixed order of rules or, if there is none, the doubling of the first eligible number (Appendix A). Problems without a must-change edit (Table A1) receive only the invariant probe.

If $c_{\mathrm{safe}}\geq \tau_{\mathrm{high}}$, the greedy answer is served immediately and no stage-2 sample is drawn. Otherwise, stage 2 draws *k* samples at temperature 0.7 and votes over k+1 answers: the greedy answer and the samples. If the plurality answer has a vote share of at least 0.6, the system serves it. Otherwise, the system declines the item and reports the reason. With k=2, serving therefore requires at least two agreeing answers out of three, and with k=4 at least three out of five.

Votes are counted under answer equivalence instead of string identity. For example, 0.5, 1/2 and \frac{1}{2} are three spellings of one value. Counting them as different answers would split the votes for one value and could reduce the number of correct answers, especially on LaTeX-heavy problems. The ballot therefore normalizes each answer and compares expressions, intervals, sets, and tuples with a symbolic comparator. Answers that the comparator cannot parse are compared as strings. The ballot checker is separate from the offline grader that assigns the correctness labels. The grader may be slower and stricter than the ballot checker.

A generation with no parseable answer casts no vote, and the share is taken over the votes cast. Of the 8845 probe generations, 25 (0.28%) yielded no parseable answer, which affected 23 items (0.51%). Such a probe still counts as one generation in Equation (1). The fingerprint records the number of answered probes of each kind, so an unanswered probe or a missing must-change edit is not treated as agreement.

Thresholds were selected backbone-out on the raw scale of Section 3.3, under two registered constraints. Serve-band precision had to be at least 0.95, and decline-band accuracy had to be at most 0.20. Both constraints were imposed on the four backbones on which a threshold was chosen, not on the backbone to which it was then applied. Table 3 lists the thresholds and the bands they produce. The threshold $\tau_{\mathrm{high}}$ is the lower bound of the immediate-serve band, and $\tau_{\mathrm{low}}$ is the upper bound of the decline band. The two-band gate uses only $\tau_{\mathrm{high}}$ and passes decline-band items to stage 2 together with the verify-band items. The three-band variant declines the decline-band items at stage 1 (Section 4.1).

### 3.5. Evaluation Protocol

Thresholds, splits, decision rules, and the adoption rule were fixed before the evaluation runs were generated and are published with the code. The adoption rule considers only the gate variants whose correct yield is at least that of the ungated model. Among these, it adopts the variant with the highest selective accuracy and breaks ties in favor of the lower generation cost. If no variant qualifies, a failure to preserve correct yield is reported.

The registration fixed the variants and this rule but no set of candidate values for the number *k* of stage-2 samples. The variants were evaluated with k=4. The gate is reported at k=2 and at k=4 (Section 4.1). The sweep over *k* in Section 4.6 uses the first *k* of four stage-2 samples drawn with fixed seeds, so each row equals a run that draws *k* samples.

### 3.6. Data and Backbones

Three benchmarks of mathematical problems were used. GSM8K is a set of English grade-school word problems [32]. The second benchmark is its Korean translation from HRM8K [33], and MATH-500 is the 500-problem test subset [34] of the MATH competition dataset [35]. From each benchmark, 300 problems were sampled with a fixed seed, and each benchmark was run with each of the five backbones. This design gave fifteen cells (backbone–benchmark pairs). An anchor check verified that every backbone received the same problems in the same positions.

The design planned 4500 evaluations, and 4497 were obtained. On three Korean items, two with qwen2-math-1.5b and one with qwen3.5-9b, the runtime returned no token count and no log-probabilities on all three attempts. The harness recorded these runtime failures before any gate decision was computed. The three items were excluded from every analysis and were not counted as wrong, declined, or uncertain. All rates use the 4497 obtained evaluations as the denominator, unless a table states a different one.

All backbones were served by the Ollama runtime (versions 0.34.0 to 0.35.1; Ollama, Palo Alto, CA, USA) with four-bit weights (Table 4). The prompt was the problem text followed by an instruction to give the final answer in \boxed{}. All cells used greedy decoding, a 2048-token limit, and the log-probabilities of the top ten tokens at each position. Stage-2 samples used the same setting at temperature 0.7.

The five backbones were chosen so that each tests one condition that the design must handle. The qwen2-math-1.5b backbone is a small model specialized for mathematics, for which the MOME score was expected to add the least information. Its accuracy also dropped on the Korean benchmark, and Section 5.4 uses this cell to examine the lower limit of the method. The gemma4-e2b and gemma4-e4b backbones are two sizes of the Gemma 4 family. They share architecture, tokenizer, and training, so a difference in results between them can be attributed to capacity rather than lineage. The qwen3.5-9b backbone (Qwen 3.5; Alibaba Cloud, Hangzhou, China) is a general multilingual model that natively supports English and Korean. The solar-10.7b backbone is a Korean-developed model of a third lineage. The five backbones therefore come from three families, and Gemma 4 and Qwen contribute two backbones each.

Across the five backbones, the accuracy of the ungated model ranged from 47.8% to 86.4%, and across the cells from 8.4% to 94.3%. The gate was therefore tested both where the backbone was strong and where it had almost no correct answers to preserve. The thinking mode was disabled on the Gemma 4 pair and Qwen 3.5, the three backbones that expose one. Because the predictor is fitted backbone-out, the five backbones are also the five held-out folds of the evaluation. Section 4.9 adds four backbones of further families, scored with a predictor and threshold fitted on these five.

### 3.7. Linear Probe on Hidden States

The MOME score was compared with a linear probe on hidden states without generating new answers. Outside the runtime, the stage-1 greedy answer of the four-bit model was passed through the same backbone with 16-bit weights in one teacher-forced forward pass. The hidden states were therefore computed by the 16-bit model on *the same answer that the gate scored*. For MATH-500, the comparison used a separate run of the two backbones with a 4096-token limit, which supplied the answers, gate decisions, and MOME scores. The linear probe is therefore an offline comparator whose signal the deployed runtime does not provide.

The hidden states were read at the position that predicts the first token of the final answer, and one linear probe was fitted for each layer. Each linear probe consists of standardization, principal component analysis (PCA) to 32 components, and $L_{2}$-regularized logistic regression. The PCA dimension was fixed in advance, and each linear probe was evaluated with stratified five-fold cross-validation. The whole pipeline was fitted inside each training fold, so no held-out item contributed to the projection in which it was scored. The best AUROC over all layers is inflated by multiple comparisons. The AUROC at the *median layer* is therefore reported as the main result, and the best layer only as an upper bound. The comparison was repeated at every layer.

Two AUROCs computed on the same items are correlated, so comparing their separate confidence intervals is not a valid test of their difference [36]. Items were resampled, and both AUROCs were computed on the same resampled set. With 2000 bootstrap replicates, this procedure gives a confidence interval for the *difference* between the two AUROCs [37]. The MOME score and the linear probe were combined by rank averaging, which fits no parameters and so cannot leak information from the held-out items into the combined score. A fitted combiner would need a further held-out split, and the comparison would then depend on that split.

## 4. Results

### 4.1. Main Comparison

The registered adoption rule selected the two-band, equivalence-voting variant. Over all evaluated items and with k=2, this gate raised selective accuracy from 69.5% to 85.8% and lowered the error rate among served answers from 30.5% to 14.2% (Table 5). Correct yield met the registered point-estimate criterion for preservation (69.5% → 69.6%).

In two further rows of the table, only the stage-1 greedy answers with the highest scores of one signal are served, up to the coverage of the gate and without stage 2. Ranking by mean token log-probability gave a selective accuracy of 76.4%, and ranking by the MOME score gave 81.9%, a gap of +5.4 p. At this coverage, mean token log-probability was the strongest of the five output statistics, and the other four reached 73.6%–75.8%. This comparison did not test every confidence estimate that a model could be made to produce. The gate with k=2 reached 85.8% at the same coverage. Its further gain of +3.9 p over the MOME score alone reflects the stage-2 ballot and its majority answers, which required extra generations.

Table 6 compares the four registered routing and voting variants at k=4, the setting of the registered evaluation. The three-band variant, which declines low-scoring items at stage 1, reached a higher selective accuracy (90.2%) than the two-band design. Its correct yield of 63.5% was below that of the ungated model, so the registered adoption rule rejected it.

Table 5 reports the gate with k=2 and k=4 stage-2 samples. Correct yield was 69.65% with k=2 and 69.62% with k=4 (one item apart), and both values reached the ungated accuracy. With k=4, selective accuracy was 88.1% (2.3 p higher than with k=2), coverage was 79.0% (81.2% with k=2), and the gate used 5.70 generations per item (4.34 with k=2). The two settings therefore trade generations against selective accuracy at the same correct yield. The preferred setting depends on this trade-off. Applied to the sweep, the registered adoption rule, which considers cost only when selective accuracy is tied, selects k=4. Both settings are reported. Unless stated otherwise, the analyses below use k=2, the setting with fewer generations. Table 7 lists the results of the gate with k=2 for each backbone.

Table A2 lists confidence intervals for the main results. Items were resampled *within* each cell at the observed cell sizes, because the design drew a fixed sample of 300 items for each cell. Resampling from the pooled items would count the differences between cells as sampling noise and widen the interval. The selective-accuracy interval was 84.7%–86.8%, above the ungated accuracy of 69.5%. Over 2000 replicates, the interval for the correct-yield difference against the ungated model was −0.6 to +0.8 p and covered zero. Because the registered adoption rule applies to the point estimate (+0.1 p), the result met the registered operational criterion for preservation but was not a formal non-inferiority result.

The change in correct yield varied across cells (Figure 3). Its interval lay above zero in 1 of 15 cells, below zero in 1 and covered zero in the remaining 13.

### 4.2. Risk–Coverage

Ranking by the MOME score gave higher selective accuracy than ranking by mean token log-probability at every evaluated coverage below full coverage (Figure 4). The widest gap was +9.0 p, near 70% coverage. The AUROC of the MOME score was 0.863 over all 4497 stage-1 greedy answers and ranged within single cells from 0.591 to 0.970, with a mean of 0.811.

### 4.3. Cost

The cost of the gate for one item, counted in generations, is

(1) $$g\;=\;\underset{\mathrm{answer}}{\underset{︸}{1}}\;+\;\underset{\mathrm{probes},\;\mathrm{fixed}}{\underset{︸}{p}}\;+\;2\times \left(1-s\right),$$

where *p* is the mean number of probes per item on that benchmark. The symbol *s* is the fraction of items that stage 1 served immediately. The value of *p* ranged from 1.92 to 1.99, below 2 because some items had no must-change edit.

The first two terms form a fixed base of about three generations, and the third applies only to items that stage 1 did not serve. The per-cell totals in Table 8 therefore differ only through *s*, the share of items that stage 1 served. The gate used 3.69 generations per item on GSM8K (en) ×gemma4-e4b, where 64.7% of items were served immediately. It used 4.98 on HRM8K-GSM8K (ko) × qwen2-math-1.5b, where 0.7% were served immediately. The mean over all items was 4.34.

As a reference, self-consistency with four samples was applied to each of the 3082 items that reached stage 2. Its vote covered five generations per item, including the greedy answer. Self-consistency returned 1813 correct answers (58.8%), and the gate returned 1814 (58.9%) on the same items. The accuracy of the ungated model on these items was 58.7%. Over all items, the gate with k=2 used fewer generations than this self-consistency baseline (4.34 against 5.00 per item), because items served immediately draw no stage-2 samples. With k=4, it used more (5.70).

### 4.4. Overhead of the Monitor and Time per Item

MOME is a routing wrapper around the backbone. It consists of the perturbation engine, the answer comparator, and a logistic score over 26 features. Apart from the generations, its processing time is the only cost that MOME adds. This overhead was measured on the committed records of the evaluation runs, without new generations (Table A3). On the slowest path, in which both probe answers were expressions, the monitor added 0.59 ms per item at the median and 5.21 ms at the 95th percentile. The scoring step took 0.004 ms at the median, and the predictor occupies 2.5 KB.

The rest of the wall-clock time is backbone generation, which depends on the backbone and the host and not on MOME. For orientation, the recorded generation times of every item were summed, without the one-time loading of the model. On the host that generated the runs, a desktop workstation with one NVIDIA GeForce RTX 5090 GPU (NVIDIA, Santa Clara, CA, USA), one greedy answer took 1.7 s at the median and 2.8 s on average. A complete gated item with the requests run one after another took 7.6 s at the median, 12.2 s on average, and 42.5 s at the 95th percentile. The mean was 4.4 times that of the greedy answer alone. The median ranged from 3.8 s on qwen2-math-1.5b to 12.9 s on qwen3.5-9b. The probes of an item do not depend on each other or on the greedy answer, and neither do the stage-2 samples. If these requests ran in parallel, the time per item would fall to the slowest request of each stage: 3.4 s at the median and 5.8 s on average. These times describe one host and one runtime and support no claim about other hosts.

### 4.5. Final State of Each Item

Table A4 lists the final state of every evaluated item and whether the relevant answer was correct. This answer is the returned answer for a served item and the ungated greedy answer for a declined item. Stage 1 served 1415 items immediately, and 97 of these answers were wrong. Stage 2 served a further 2237 items, of which 423 were wrong. Of the greedy answers withheld by the 845 declines, 757 would have been wrong and 88 would have been correct.

An item-by-item trace shows that stage 2 replaced 116 wrong greedy answers with correct ones and 23 correct ones with wrong ones. Declining withheld the 88 correct greedy answers counted above. The net difference, 116 − 23 − 88 = +5 items, corresponds to the +0.1 p in Table 5.

In this evaluation, the two mechanisms of the design contributed unequally. Most declined greedy answers would have been wrong, so the gain in selective accuracy came from declining. Stage 2 changed answers in both directions, and its small net gain of correct answers offset the small loss from declining. Correct yield therefore stayed equal to that of the ungated model.

### 4.6. Odd and Even Vote Counts

The results did not change monotonically with the number *k* of stage-2 samples (Table 9). With k=3, the ballot has four votes, and a two-to-two tie gives a vote share below the 0.6 serving threshold, so the gate declines a tied item. Correct yield at k=3 changed by −3.2 p relative to k=2. This drop follows from two choices in the serving rule of this study, the 0.6 threshold and the decline of ties, and is not a general property of voting. Under these choices, the parameter that mattered most was ballot parity, whether the ballot has an odd or an even number of votes. The smallest *k* that gives an odd ballot is k=2, and the next is k=4.

### 4.7. Sensitivity to the Threshold and the Vote Rule

Table A5 varies the two decision parameters of the gate with k=2, the threshold $\tau_{\mathrm{high}}$ and the stage-2 vote rule, on the registered MOME scores. The threshold $\tau_{\mathrm{high}}$ of every backbone was shifted by the same amount. With a shift of −0.10, coverage was 82.6% and selective accuracy was 84.9%. Correct yield changed by +0.58 p against the ungated model, at 3.73 generations per item.

Raising the threshold sends to stage 2 some items that stage 1 served in the evaluation and that therefore have no stage-2 samples. A shift of +0.01 already moved 299 items, because many scores lay just above the thresholds. Their outcome was estimated from the observed stage-2 items closest to the serve band (caption of Table A5). Over the whole range of shifts, the estimated change in correct yield stayed between −0.49 and +0.58 p, and selective accuracy between 84.9% and 86.0%. For positive shifts, this result depends on the estimate. The bounds in the last column of the table are wide, and the records cannot narrow them without new stage-2 samples.

The vote rule had a larger effect than the threshold. Requiring unanimity instead of two agreeing votes raised selective accuracy to 92.2%. It also lowered coverage to 66.0% and changed correct yield by −8.67 p. Serving the greedy answer when no two votes agreed gave a coverage of 81.9%, a selective accuracy of 85.0% and a change in correct yield of +0.13 p. Requiring agreement therefore traded little correct yield for selective accuracy, and requiring unanimity changed correct yield by −8.67 p.

### 4.8. Fitting Without the Scored Problems, Model Family or Benchmark

In the registered evaluation, the predictor and the thresholds for a backbone were fitted on the other four backbones, which answered the same problems. Table 10 repeats the fit with three stricter separations on the fingerprint features of the evaluation. In every row except the first, $\tau_{\mathrm{high}}$ was selected again with the registered procedure on the fitting data only. Each separation is compared with the backbone-out fit of the second row, which reproduces the registered scores (AUROC 0.863).

*Thresholds on the held-out backbone.* The serve-band precision of at least 0.95 was required only on the four fitting backbones (Section 3.4). Table 3 shows that it was not met on three of the five backbones: solar-10.7b (0.834), qwen2-math-1.5b (0.903) and gemma4-e4b (0.9498, shown as 0.950 in the table). The two largest shortfalls occurred on the two backbones with the lowest ungated accuracy, 47.8% and 52.2%, against 77.4% to 86.4% for the other three. On solar-10.7b and qwen2-math-1.5b, no threshold reached a precision of 0.95 unless it served at most 0.4% of the items. Over all backbones, 97 of the 1415 answers served at stage 1 were wrong, a precision of 0.931. In this evaluation, the thresholds therefore did not carry the precision target over to a backbone with a much lower accuracy than the fitting backbones. In each of the stricter separations below, the stage-1 precision was also below 0.95 (Table 10).

*Disjoint problems.* The problems were split into five folds, and the English and Korean versions of a problem were kept in the same fold. For each held-out backbone and fold, the predictor and the threshold were fitted on the items of the other four backbones whose problems were in the other folds. Every scored answer was then new to the fit in both backbone and problem. The AUROC was 0.859 (95% interval 0.846–0.872), a change of −0.003 (−0.005 to −0.002) against the backbone-out fit on the same features. The gate reached a coverage of 81.1% and a selective accuracy of 85.8%, and correct yield changed by +0.02 p (95% interval −0.63 to +0.67). The re-fitted score sent 252 items without stage-2 samples to stage 2. If all of them were declined, the change would be −5.11 p, and if all were served with the greedy answer, it would be +0.16 p.

*Held-out model family.* In the registered fit, the predictor for one Gemma backbone was fitted with the other Gemma backbone, and likewise for Qwen. When the whole family was held out, the AUROC was 0.863, a change of +0.001 (−0.002 to +0.004). The AUROC was 0.837 on the Gemma backbones, against 0.848 with the backbone-out fit, and 0.892 on the Qwen backbones, against 0.875. Correct yield changed by +0.29 p, with 6 items without stage-2 samples (+0.20 to +0.29 p under the two extreme assumptions).

*Held-out benchmark.* The English and Korean GSM8K benchmarks share their problems, so the held-out benchmark was either both of them or MATH-500. The AUROC fell to 0.835, a change of −0.028 (−0.034 to −0.022). The thresholds did not transfer, and the precision of the answers served at stage 1 was 86.7%. The re-fitted score also sent 533 items without stage-2 samples to stage 2, so the change in correct yield was bounded only between −10.99 and +0.09 p.

In summary, the discrimination of the MOME score changed little with unseen problems or an unseen model family, and the estimated change in correct yield stayed near zero. This estimate rests on items without stage-2 samples (252 for unseen problems). On an unseen benchmark, the score lost part of its discrimination, and the thresholds had to be selected again.

### 4.9. Backbones of Further Families

Four further backbones were evaluated (Table 11). They belong to the Llama 3.2, Phi-4, EXAONE 3.5, and Granite 3.3 families, whose developers are absent from the main evaluation. In runtime, Llama 3.2 has the same architecture type as solar-10.7b, which was built on the Llama 2 architecture. It differs from that backbone in developer, training data, and tokenizer. Each backbone answered the same 900 problems with the same runtime, prompt, decoding settings, and probes, and received four stage-2 samples for every item. The predictor and $\tau_{\mathrm{high}}$ were fitted once on all 4497 items of the five backbones with the registered procedure. Both were frozen before the four backbones were run, with $\tau_{\mathrm{high}}=0.911$. No item of the four backbones entered the fit, and the evaluation criteria were registered before their answers were generated. The registered criteria were an AUROC difference above zero on each backbone, a gain in selective accuracy whose 95% interval excluded zero, and a change in correct yield over the four backbones with a point estimate of at least zero and a lower 95% bound of at least −2.0 p.

*Discrimination.* On each of the four backbones, the MOME score separated correct from wrong answers better than mean token log-probability. Its AUROC was between 0.782 and 0.852, against 0.547 to 0.662. The item-paired differences were between 0.124 and 0.276, and all four 95% intervals excluded zero. Over the four backbones, the AUROC was 0.829 against 0.629.

*Stage-2 samples at temperature 0.7.* At the setting of the main evaluation and with k=2, the gate raised selective accuracy over the four backbones by +17.0 p (95% interval +15.8 to +18.3). Correct yield changed by −1.81 p, with a 95% interval of −2.72 to −0.89 (Table 12). The loss came mainly from llama3.2-3b, with −6.11 p (95% interval −8.44 to −4.00); phi4-mini changed by −1.33 p (−3.11 to +0.67). The two larger backbones stayed near the ungated level, exaone3.5-7.8b with +0.67 and granite3.3-8b with −0.44. On the items sent to stage 2, the samples of llama3.2-3b were correct for 39.8%, against 47.6% for its greedy answers. For phi4-mini, the two rates were 59.9% and 63.1%. On the five backbones of the main evaluation, the difference lay between −1.1 and +2.5 p. In this evaluation, the backbones whose samples were less accurate than their greedy answers were the backbones on which correct yield fell.

*Stage-2 samples at temperature 0.3.* The stage-2 samples of all nine backbones were also drawn at temperature 0.3, with the same prompts and seeds. This temperature was registered after the results at 0.7 were known and was not tuned. At 0.3, the accuracy of the samples differed from that of the greedy answers by −1.2 to +2.8 p on the nine backbones. Over the four further backbones, correct yield changed by −0.19 p with k=2, with a 95% interval of −1.00 to +0.61. With k=4, it changed by −0.03 p, with an interval of −0.92 to +0.86. On the five backbones of the main evaluation, the changes were +0.62 and +0.60 p. The lower temperature cost selective accuracy. With k=2, it fell from 85.0% to 81.7% over the four further backbones and from 85.8% to 84.4% on the five. The gain in selective accuracy over the ungated accuracy stayed above zero on each of the four backbones in every configuration, with a lowest interval bound of +3.6 p.

*Number of samples.* As on the five backbones, k=4 gave a higher selective accuracy than k=2 at both temperatures and used more generations. Per backbone, the difference in correct yield between k=4 and k=2 lay between −0.89 and +2.11 p and changed sign; over the four backbones, it was +0.39 p at 0.7 and +0.17 p at 0.3.

In summary, the MOME score kept its advantage over the output statistics on four further families without being fitted on them. Whether the gate kept correct yield at the ungated level depended on the stage-2 temperature. At 0.7, it kept correct yield on the five backbones and near it on the two larger further backbones, but not on the two smallest. At 0.3, correct yield over the four further backbones was −0.19 p from the ungated level, at a lower selective accuracy. For the registered criterion, the two smallest backbones changed by −3.72 p at 0.7 and −0.50 p at 0.3, and the two larger ones by +0.11 p at both temperatures. The discrimination and selective-accuracy criteria were met in every configuration. The correct-yield criterion was not met at 0.7 (−1.81 p, interval −2.72 to −0.89) or at 0.3, where the point estimates were −0.19 p with k=2 and −0.03 p with k=4.

### 4.10. Contribution of the Feature Groups

Table 13 shows the contribution of each part of the fingerprint. The predictor was re-fitted backbone-out with one group of features alone and without one group. All features together reached an AUROC of 0.863.

The invariant probe accounted for most of the discrimination of the score. Its four features alone reached an AUROC of 0.759, and without them the AUROC fell to 0.764 (95% interval of the change −0.110 to −0.088). The ten features of the must-change probe alone reached 0.516 (0.497–0.533), close to chance. Without them, the AUROC was 0.854, with a 95% interval of the change of −0.013 to −0.004. The selective accuracy at the coverage of the gate did not change measurably (95% interval of the change −0.3 to +0.1 p). In this evaluation, a must-change violation occurred for 11.4% of the wrong and 10.3% of the correct greedy answers that received a must-change probe. An invariant violation occurred for 71.8% of the wrong and 8.9% of the correct answers. On these benchmarks, the failure that only a must-change probe can detect (Section 1.4) was not frequent enough to raise the AUROC. The three statistics of the original answer, among them mean token log-probability, reached 0.647 alone. Without them, the AUROC was 0.844 (95% interval of the change −0.026 to −0.012). The two probe groups together reached 0.801 alone, and without both of them the score fell to 0.754.

### 4.11. English and Korean Versions of the Same Problems

The English and Korean cells contain the *same* 300 problems with one-to-one item indices and numerically equal gold answers. The results were therefore paired by item, which keeps each arithmetic problem fixed across the two languages. Table 14 reports, for each language, how often the invariant edit changed the answer. Translation, tokenization, and the amount of training data in each language vary together with the language, and this analysis does not separate them.

Overall, the rate of answer changes increased from 12.2% in English to 31.2% in Korean, a paired difference of +19.0 p (95% interval +16.4 p to +21.6 p). The increase appeared in 3 of the 5 backbones. Descriptively, the two backbones without the increase were the two with the highest Korean accuracy. The largest increase, +68.1 p, occurred on qwen2-math-1.5b, whose accuracy dropped from English to Korean. With so few backbones, the relation between Korean accuracy and the size of the increase is reported as an untested observation. Its direction agrees with a report that relates the gap between English and other languages to the model’s comprehension of the input language rather than to its reasoning [33].

### 4.12. Comparison of the MOME Score with the Linear Probe

Table 15 compares three signals: the linear probe on hidden states, the MOME score, and their combination by rank averaging. All three signals were scored on the same items with the same outcome labels, 897 items on gemma4-e4b and 899 on qwen3.5-9b. This set (the stratum) contains every item whose greedy answer was teacher-forced without error. It covers all evaluated items on qwen3.5-9b. On gemma4-e4b, the forward pass failed for an unrecorded reason on three items, all labeled wrong.

The outcome label records whether the answer that the gate would deliver was correct. This answer is the majority answer for items served at stage 2 and the greedy answer for all other items. The label therefore differs from the correctness of the greedy answer when stage 2 replaced a wrong greedy answer with a correct one. The label does not record whether the problem was hard. The MOME score is the score $c_{\mathrm{safe}}$ that the gate used, and neither the gate nor its score was re-fitted for the comparison.

In this stratum, the AUROC of the linear probe at the median-AUROC layer was 0.756 on gemma4-e4b and 0.832 on qwen3.5-9b, above chance. For comparison, the same items were scored with the same outcome label by mean token log-probability, the strongest output statistic on all items (Section 1). Its AUROC was 0.691 on gemma4-e4b (95% interval 0.635–0.750) and 0.777 on qwen3.5-9b (0.723–0.827). The item-paired difference between the linear probe and mean token log-probability was +0.065 (−0.013 to +0.142) on gemma4-e4b and +0.054 (−0.010 to +0.116) on qwen3.5-9b.

Rank averaging of the MOME score and the linear probe raised the AUROC above that of the linear probe alone by +0.041 on gemma4-e4b and +0.040 on qwen3.5-9b. In both cases, the item-paired 95% interval of the gain excluded zero. Across the layer sweep, the interval of the gain excluded zero at 35 of 43 layers on gemma4-e4b and at 33 of 33 layers on qwen3.5-9b (Figure 5). When each signal was used alone, the paired bootstrap did not distinguish the linear probe from the MOME score. Their difference in AUROC was +0.015 on gemma4-e4b and −0.024 on qwen3.5-9b. Both intervals covered zero, and the signs were opposite.

### 4.13. Confident Answers Flagged by the Linear Probe

Figure 6 plots the score of the linear probe against the mean token log-probability of each item. On gemma4-e4b, 15 items were in the quadrant where the linear probe predicted a wrong answer and the mean token log-probability was high. Of these items, 5 (33%) were wrong, compared with 12% of all items in this comparison, and the gate declined 1 of them. This quadrant is reported as an observation because it met exactly the minimum of 15 items that the registered analysis rule requires. On qwen3.5-9b, the same quadrant contained a single item, which was not assessed.

## 5. Discussion

### 5.1. Contribution of the Design Elements

In this evaluation, the MOME score ranked answers better than every output statistic of the runtime (Section 1), and the gate kept correct yield at the ungated level with two and with four stage-2 samples. The preservation of correct yield is attributed mainly to three properties of stage 2. The first is ballot parity: because the gate declines every tie, a third stage-2 sample can reduce correct yield compared with two samples (Section 4.6). Work on self-consistency treats the sample count as a smooth trade-off between cost and quality [11]. The result of Section 4.6 suggests that the trade-off also depends on ballot parity when ties are declined.

The second property is answer equivalence (Section 3.4). Counting votes by answer equivalence instead of string match added +1.3 p of correct yield in the two-band design at k=4 (Table 6). The two-band design has the same purpose, which is to avoid declining correct answers. A low MOME score shows that a probe answer did not change or did not stay the same as its rule requires. The score does not identify the correct answer. The gate therefore sends low-scoring items to stage 2, and in this evaluation the choice preserved correct yield.

The third property is the quality of the stage-2 samples. A vote can keep a correct greedy answer only if the samples reproduce it. Samples drawn at temperature 0.7 matched the accuracy of the greedy answer on the five backbones. On the two smallest further backbones, they fell below it, and correct yield dropped (Section 4.9). Samples drawn at 0.3 matched it on all nine backbones, at a lower selective accuracy. In this evaluation, the stage-2 temperature that kept correct yield near the ungated level depended on the backbone; a procedure to choose it for a new backbone was not tested.

### 5.2. MOME Score and Linear Probe

The above-chance AUROC of the linear probe (Section 4.12) agrees with reports that structured information can be recovered from the internal representations of transformers [28,29,30]. The gain from adding the MOME score concerns prediction on the evaluated items, and no information-theoretic claim is made about the content of either signal. The paired bootstrap did not distinguish the AUROCs of the two signals alone. With about nine hundred items per backbone, this result does not demonstrate equality. This study therefore claims neither that behavioral testing is better than introspection nor that the two are equal.

These results support the design of MOME because the linear probe cannot be applied in the deployment setting of this study. The runtime does not expose hidden states, and no correctness labels are available at deployment to fit a linear probe. The MOME score needs only additional generations of the backbone, and it ranks answers better than the output statistics that the runtime provides (Section 1). The claim here is that the MOME score can be used in deployment and complements the linear probe. This study does not determine whether the linear probe encodes “this answer is wrong” or “this problem is hard”. The same question applies to what the MOME score adds.

### 5.3. Use of the Procedure in Other Domains

A study in another domain can reuse the procedure of Section 1.2 but not the fitted components, and it would have to supply three things. First, it needs edits whose effect on the correct answer can be derived before the model answers, at least one keeping and one changing that answer. Second, it needs a rule that decides whether two answers are the same. In arithmetic, this rule compares values, whereas in other domains it can be uncertain, and its errors would enter the fingerprint and the vote. Third, it needs labeled items from several backbones to fit the score backbone-out and to select the thresholds. The two kinds of violation, the two-stage policy, the adoption rule, and the correct-yield criterion then apply without change. The observations on the direction and size of a change (Section 3.3) are specific to arithmetic. The following two examples are untested hypotheses.

*Code generation.* The answer is a program, and the rule for comparing answers is execution. Two programs give the same answer if they produce the same outputs on a fixed set of inputs. The gate can generate these inputs without a reference solution. An invariant edit restates the task without changing its specification, for example by renaming a function or by repeating a constraint already given. A must-change edit alters one checkable condition, for example *return the largest* instead of *return the smallest*, a strict instead of a non-strict bound, or a shifted range. The outputs must then differ on at least one input that the edit concerns. A backbone that returns the same program has ignored the changed condition, as in arithmetic. This domain is expected to be the closest to arithmetic, because execution gives a definite comparison of answers.

*Fact checking.* The answer is a verdict on a claim, such as *supported* or *refuted*, and the rule for comparing answers is equality of the verdicts. An invariant edit paraphrases the claim or reorders its clauses. A must-change edit negates the claim or replaces an entity, a date, or a quantity with one that the evidence contradicts, so that the correct verdict must change. The expected effect is less certain than in arithmetic. A negation can produce a claim that the evidence neither supports nor refutes, and a replaced entity can be correct by chance. Fact checking is therefore expected to produce more defective edits than arithmetic did in this study. Their effect on the score would have to be measured before the gate is used.

### 5.4. Limitations and Threats to Validity

#### 5.4.1. Restriction to Mathematical Problems

The evaluation was restricted to mathematical word problems so that the correctness of every answer could be measured exactly (Section 1.2). This restriction is the main limit of the study. None of the fitted components and none of the measured gains were tested on tasks with open or judged answers.

#### 5.4.2. Low Base Accuracy

The one cell with a correct-yield interval below zero (Figure 3) combined Korean problems with qwen2-math-1.5b and had the lowest base accuracy, 8.4%. The gate reached a correct yield of 4.4% (−4.0 p) at 19.8% coverage. A selective policy can serve only the correct answers that the backbone generates, and when these are rare, declining removes most of them. Another cell, with an ungated accuracy of 17.7%, had no interval below zero. Near-zero base accuracy is therefore a working guideline for when not to use the gate, not a tested threshold.

#### 5.4.3. Truncation

The greedy answer reached the token limit on 234 items (5.2%), and the ungated model was correct on 4.7% of them. Without these items, the ungated accuracy rose from 69.5% to 73.1%, and the gate served 84.6% of the remaining items with a selective accuracy of 86.1%. Its correct yield differed from the ungated accuracy by −0.3 p, against +0.1 p on all items. On the truncated items, the gate returned 11.5% correct answers against 4.7% for the ungated model, so the preservation of correct yield on all items depended on them (see also the 4096-token run below).

#### 5.4.4. Coverage of the Rules

The perturbation engine cannot edit every problem, and on MATH-500 it produced no must-change edit for 23 of 300 items. The MOME score of these items used the invariant probe only, and the missing edit entered the score as a feature.

#### 5.4.5. Validity of the Edits

The edited problems were not audited one by one, and no edited problem was solved by a person or by a second model. Rule certainty is an annotation of the rule type (Table 2 and Table A1), not a measured validity rate. Three defects are known. The Korean comparative substitution does not adjust the particle or verb ending after the substituted word. The result can be ill-formed. A reversed LaTeX inequality can leave no valid value or no unique answer, and a doubled quantity that does not enter the computation leaves the correct answer unchanged.

When the model keeps its answer, a defective edit can send the item to stage 2 at extra cost. When the model changes its answer, a wrong answer can keep a higher score than a valid edit would give it. How often each case occurred was not measured. The serve-band precision in Table 3 was measured with these defects present. The comparison in Section 4.11 used only the invariant probe, which these defects do not affect.

#### 5.4.6. Scope of the Linear-Probe Comparison

The comparison with the linear probe covered two backbones and one problem domain. The results of Section 4.12 are an initial observation.

#### 5.4.7. No New Problems or Benchmark at Generation Time

The re-analysis of Section 4.8 used the committed generations, so all scored problems came from the same three benchmarks and the same sample of 300 problems each. The gate was not run on newly generated problems, and the outcomes of items that were re-fitted and sent to stage 2 without samples were estimated, not observed.

#### 5.4.8. Model Families

The main evaluation used backbones of three families (Section 3.6). Holding out a whole family changed the AUROC by +0.001 (Section 4.8). Backbones of four further families kept the advantage of the score over the output statistics (Section 4.9). They answered the same problems as the main evaluation, and on the two smallest of them, correct yield depended on the stage-2 temperature.

#### 5.4.9. Number of Stage-2 Samples

The registration fixed no candidate values of *k*, and its adoption rule, applied to the sweep, selects k=4 (Section 4.1). Both k=2 and k=4 are reported side by side. They reached the same correct yield within one item, and k=4 gave a higher selective accuracy at more generations. Most analyses of Section 4.3, Section 4.4, Section 4.5, Section 4.6, Section 4.7 and Section 4.8 use k=2.

#### 5.4.10. Dependence on the Token Limit

A separate registered run tested the gate with k=2 on three MATH-500 cells with a 4096-token limit instead of 2048 tokens. It required correct yield to reach the ungated accuracy of that run on two cells. The criterion was met on qwen3.5-9b, at +0.3 p, and not on gemma4-e2b, at −0.7 p. On the third cell, gemma4-e4b, the change was −2.0 p. With the larger limit, fewer answers were truncated, and the ungated accuracy rose to 78.7% on these 900 items. The gate reached a correct yield of 77.9%, a change of −0.8 p. Part of the gain at 2048 tokens therefore came from detecting truncated answers (Truncation, above), so the MATH-500 results depend on the token limit. The two limits were compared on three cells only.

#### 5.4.11. Re-Selection of Thresholds

The thresholds were selected for the runtime, the four-bit weights, the generation setting, and the benchmarks of Section 3.6. On a held-out benchmark, they did not keep their serve-band precision. A deployment that changes any of these should select the thresholds again with the registered procedure. On the two held-out backbones with the lowest accuracy, the precision target was not met (Section 4.8). A deployment should therefore measure serve-band precision on a small labeled sample of a new backbone before it relies on stage 1.

#### 5.4.12. Correctness Labels and Preregistration

The correctness labels come from the automatic grader of Section 3.4, and they were not checked by hand. Grader errors would therefore enter both the labels used to fit the score and the labels used for evaluation. The preregistration is a versioned internal ledger and was not filed with an external registry.

## 6. Conclusions

MOME was built for a runtime that returns tokens and log-probabilities but no hidden states, where only further generations of the same backbone can add evidence. In this evaluation, the answers to rule-based edits ranked correct above wrong answers better than every output statistic of the runtime (AUROC 0.863 against at most 0.734), and the invariant edit supplied most of this ranking. Sending low-scoring items to a vote kept the number of correct answers at the ungated level with two and with four stage-2 samples. Four samples gave 2.3 p more selective accuracy at 5.70 instead of 4.34 generations per item. With two samples, the gate used fewer generations than four-sample self-consistency (5.00 per item) and returned as many correct answers on the items it verified (1814 against 1813).

The ranking changed little when the scored problems or the scored model family were left out of the fit, and it stayed ahead of mean token log-probability on backbones of four further families. On an unseen benchmark, the thresholds had to be selected again. On the two smallest further backbones, correct yield changed by −3.72 p with stage-2 samples at temperature 0.7 and by −0.50 p at 0.3, and over the main evaluation the preservation of correct yield depended on items whose greedy answer was truncated. Must-change edits that alter the answer more reliably, an audit of edit validity and a rule for choosing the stage-2 temperature of a new backbone remain to be developed. Within these limits, edits whose effect on the correct answer is known in advance gave small models a check on their own answers that needed neither labels nor access to their internals.

## Figures and Tables

**Figure 1:**
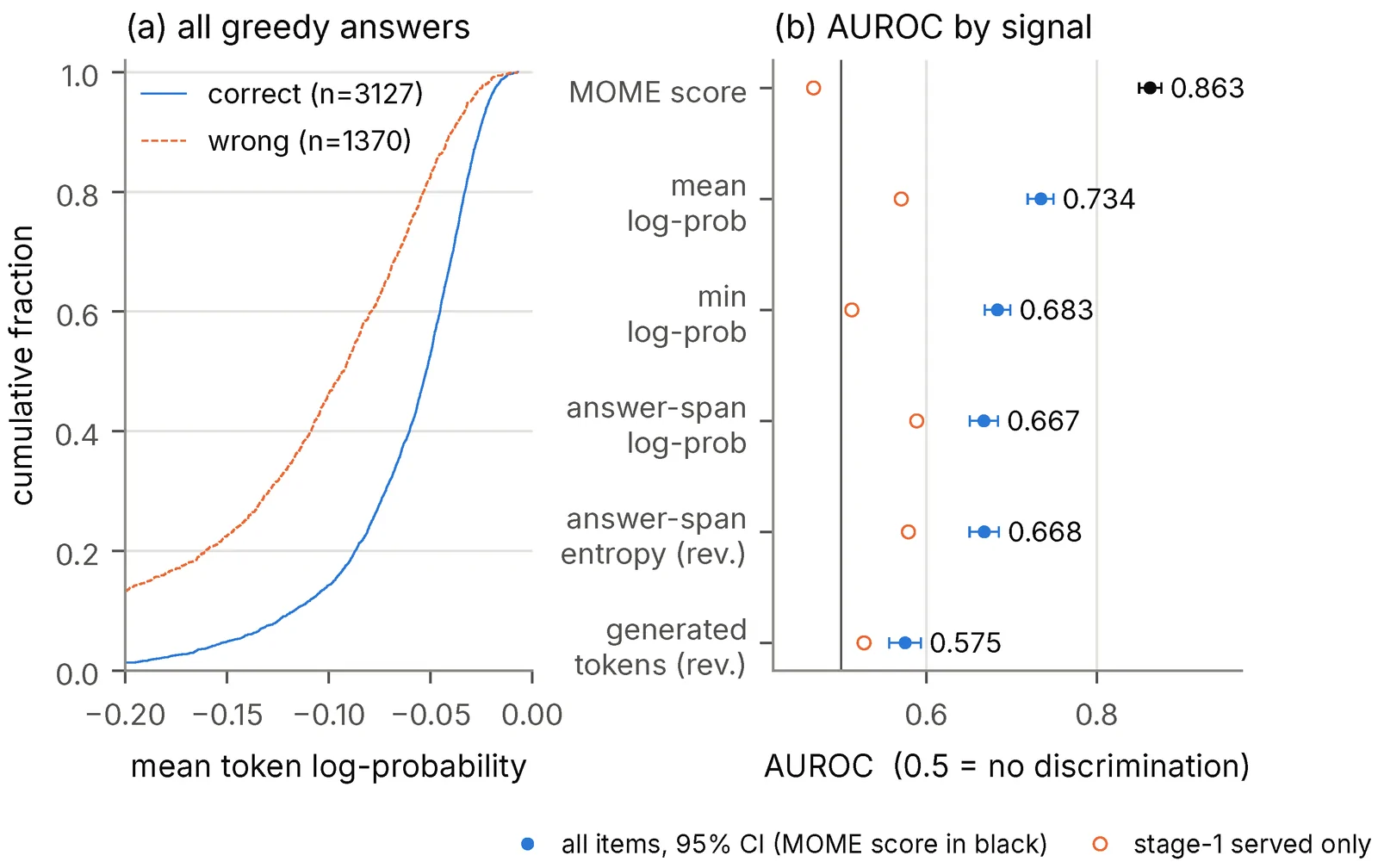
Output statistics exposed by the runtime and the MOME score on all 4497 greedy answers. (**a**) Empirical cumulative distributions of mean token log-probability for correct and wrong answers. The horizontal axis is cut at −0.20; each curve starts at the share of answers below this value. (**b**) AUROC between correct and wrong answers with the 95% interval (filled markers; 2000 bootstrap replicates resampled within cells); the MOME score is shown in black and the output statistics in blue. Entropy and length are ranked in reverse. Open markers show the same signals on the 1415 answers that stage 1 served immediately, a subset selected by a cutoff on the MOME score and therefore range-restricted.

**Figure 2:**
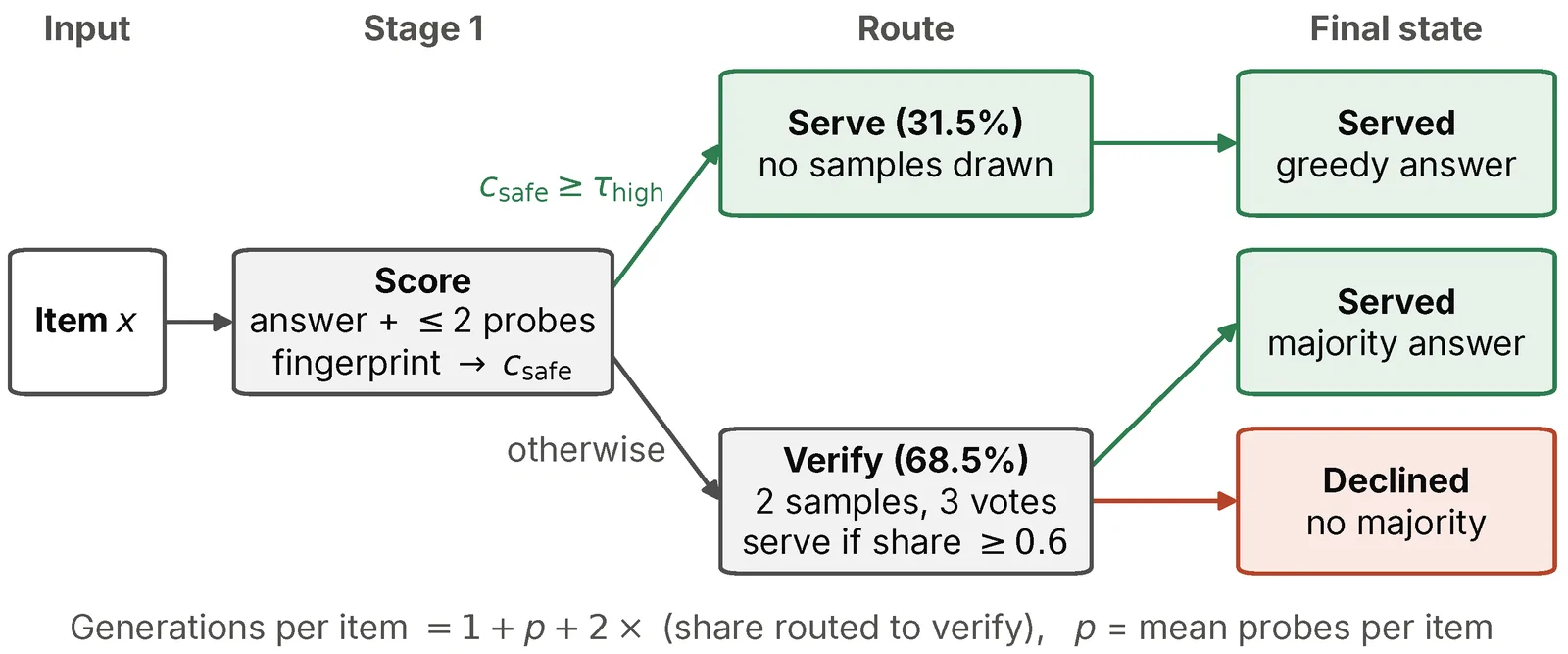
The two-stage gate and the measured flow of items with k=2. Stage 1 generates one answer and up to two probes for every item. Only the items that stage 1 does not decide go to stage 2, which draws *k* self-consistency samples. Every item ends as served (green) or declined (red), and gray marks the processing steps. The mean number of probes per item, *p* in Equation (1), was between 1.92 and 1.99, because some problems have no must-change edit. Percentages are pooled over all evaluated items, and Section 4.3 reports the shares for the cheapest and the costliest cells.

**Figure 3:**
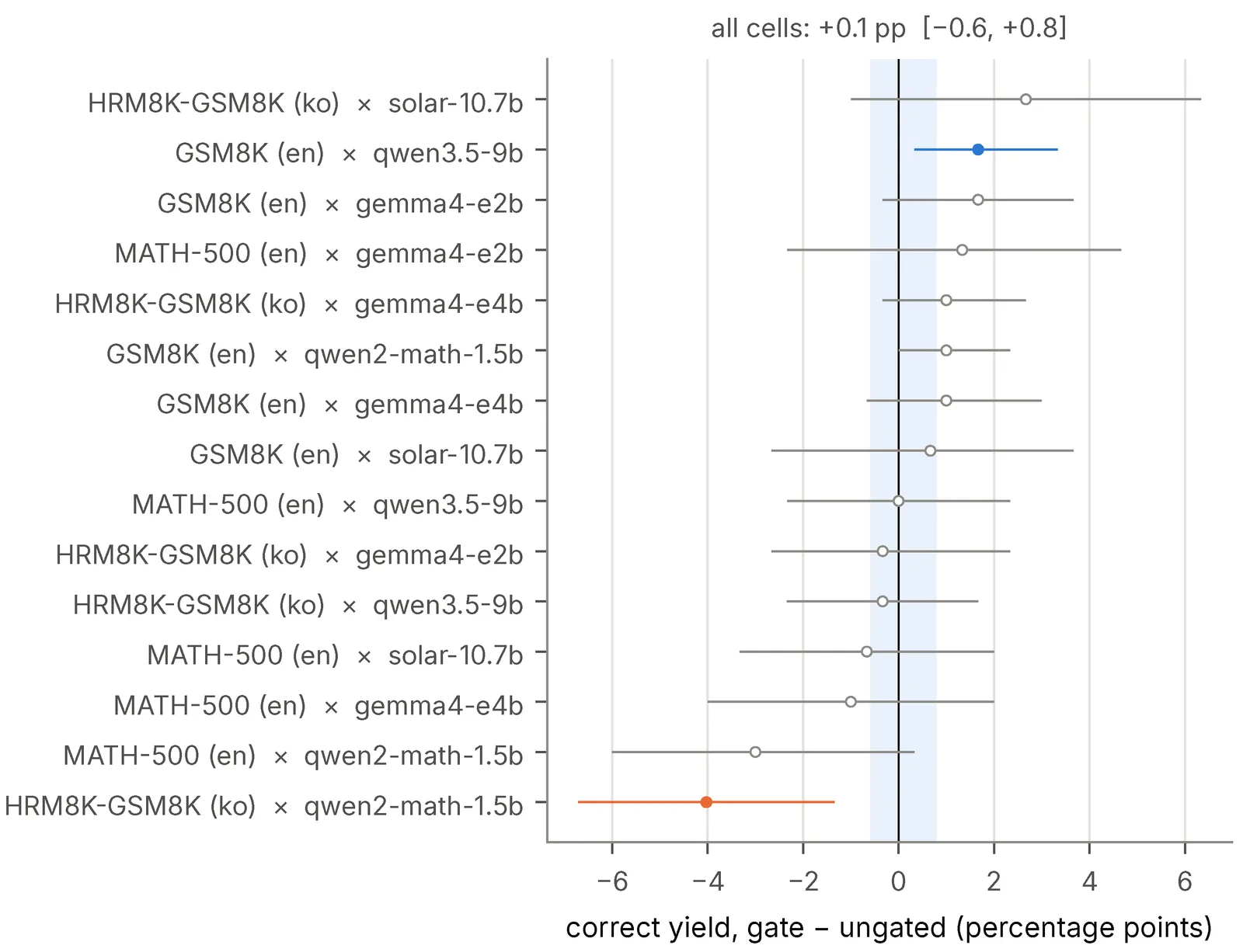
Correct yield of the gate minus that of the ungated model in each cell, with the 95% interval from resampling items within the cell (2000 replicates). The shaded band shows the interval for all cells together, and filled markers denote cells whose interval excludes zero. Blue and orange filled markers denote cells whose interval lies above and below zero, respectively, and gray open markers denote cells whose interval covers zero. Cells are sorted by the difference.

**Figure 4:**
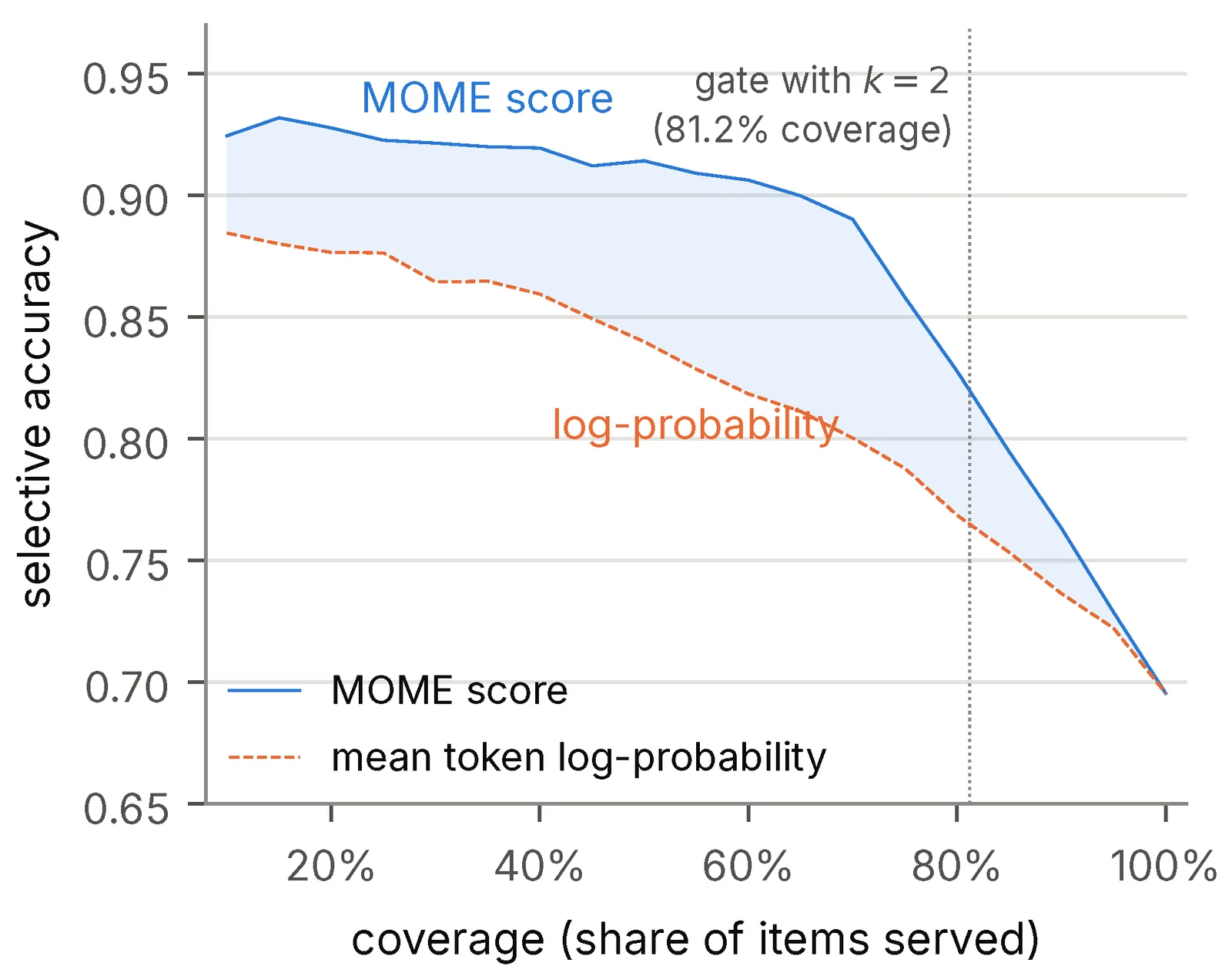
Risk–coverage curves for ranking by the MOME score and by mean token log-probability on the same items. The MOME-score curve lies above the log-probability curve at every evaluated coverage below full coverage, and the shaded area is the gap. The curves coincide at full coverage, where no ranking can change accuracy.

**Figure 5:**
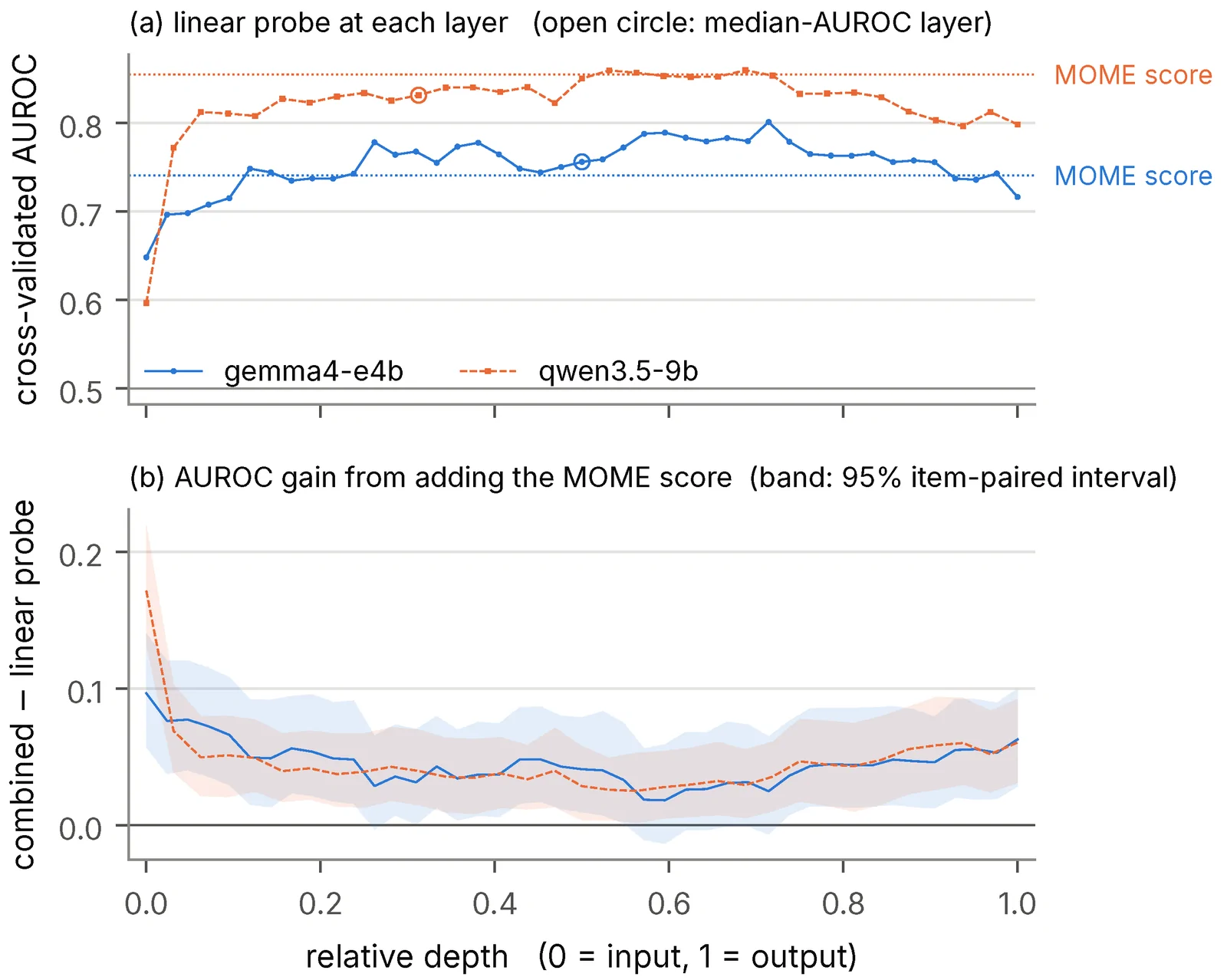
Linear probe and MOME score at each layer. (**a**) Cross-validated AUROC of the linear probe on hidden states at each layer. The open circle marks the median-AUROC layer, the layer whose AUROC is the median over all layers, used for the main result. The dotted line shows the AUROC of the MOME score on the same items. (**b**) AUROC gain from combining the MOME score with the linear probe at each layer, with item-paired 95% intervals (shaded areas). In both panels, blue denotes gemma4-e4b and orange qwen3.5-9b.

**Figure 6:**
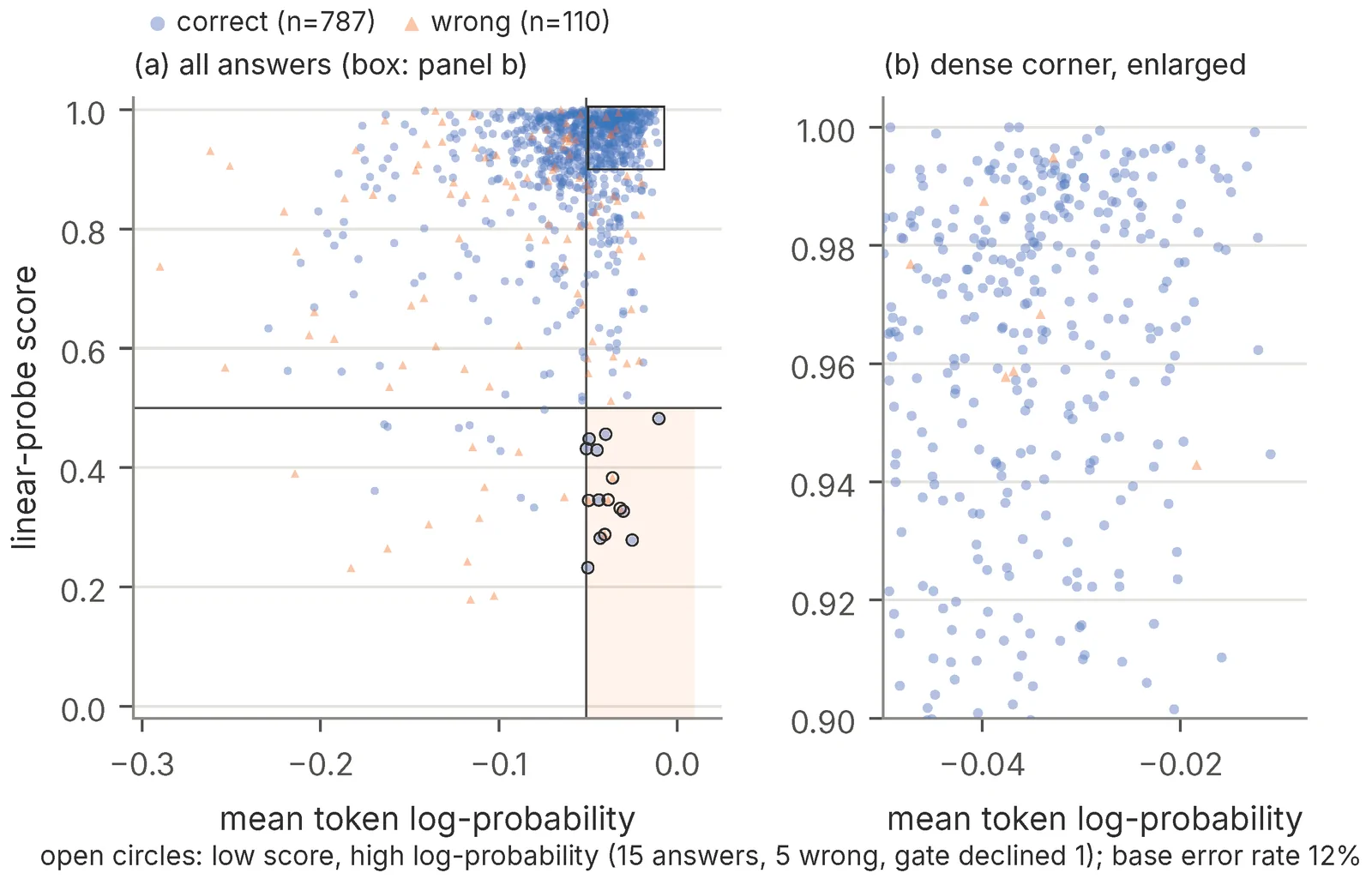
Linear-probe score against mean token log-probability on gemma4-e4b. The linear probe is an offline comparator on the 16-bit model. The shaded quadrant contains the 15 items whose answers the linear probe predicted to be wrong and whose mean token log-probability was high. Of these, 5 were wrong (base error rate 12%), and the gate declined 1; open circles mark these items. The vertical line is the median mean token log-probability on this backbone, and the horizontal line is a linear-probe score of 0.5; the score is the probe’s estimated probability that the answer is correct. (**a**) All answers. (**b**) The dense corner marked by the box in (**a**), enlarged.

**Table 1:** The invariant edit and the must-change edit of one problem (GSM8K problem 6). The invariant edit adds a redundant clause, so the correct answer stays 260. The must-change edit reverses one comparison, so Toulouse has 40 sheep instead of 160 while Charleston still has 80, and the correct answer changes from 260 to 140. Both edits come from rules that the English rule pack marks as high certainty.

Variant	Problem Text	Expected
Original	Toulouse has *twice* as many sheep as Charleston. Charleston has 4 times as many sheep as Seattle. How many sheep do Toulouse, Charleston, and Seattle have together if Seattle has 20 sheep?	—
must-change	Toulouse has *half* as many sheep as Charleston. …(unchanged)	140 (was 260)
must-hold	…if Seattle has 20 sheep? *(All quantities are exactly as stated.)*	260 (unchanged)

Italics mark the words that the edits change or add.

**Table 2:** Example edits from each rule pack, with the correct answer before and after the edit. The English and Korean edits are taken from benchmark problems: GSM8K problem 6 is the problem of Table 1, and HRM8K problem 6 is its Korean version. GSM8K problem 0 reads *Janet’s ducks lay 16 eggs per day. She eats three for breakfast every morning and bakes muffins for her friends every day with four. She sells the remainder at the farmers’ market daily for $2 per fresh duck egg. How much in USD does she make every day at the farmers’ market?* The LaTeX row is a constructed problem, because MATH-500 items are not reproduced here. In every row with a must-change edit, the correct answer changes, although the rules do not check this change (Section 3.2). Korean problems receive the redundant clause in Korean, and Section 5.4 reports the known defects of the rules.

Pack	Rule	Edit	Answer	Certainty
English	comparison reversal	GSM8K problem 6: *twice as many*→ *half as many*	260 → 140	high
English	quantity scaling	GSM8K problem 0: *16 eggs* → *32 eggs*	18 → 50	medium
Korean	comparison reversal	HRM8K problem 6: *twice* → *half* (Korean surface form)	260 → 140	high
LaTeX	inequality direction	smallest positive integer *n* with $n^{2}\geq 50$ → $n^{2}\leq 50$	8 → 1	high
all three	redundant clause	append *(All quantities are exactly as stated.)*	unchanged	high

Italics mark the text before and after each edit.

**Table 3:** Backbone-out thresholds and the stage-1 score bands that result from them. **The two-band gate serves only items in the Serve band at stage 1. Items in the Verify band and in the Decline band both go to stage 2.** The Decline band is reported for diagnosis and for the three-band variants of Section 4.1. The thresholds for each backbone were selected on the other four backbones. The selection used the registered constraints of serve-band precision ≥0.95 and decline-band accuracy ≤0.20. These constraints apply to the four selection backbones only. Serve, Verify, and Decline are the shares of items in each band. Serve prec. is the serve-band precision measured on the held-out backbone.

Backbone	$\tau_{\mathrm{high}}$	$\tau_{\mathrm{low}}$	Serve	Verify	Decline	Serve Prec.
gemma4-e2b	0.919	0.537	34.3%	40.1%	25.6%	0.958
gemma4-e4b	0.904	0.540	50.8%	32.9%	16.3%	0.950
qwen3.5-9b	0.910	0.519	18.2%	66.5%	15.2%	0.994
solar-10.7b	0.921	0.351	21.4%	51.7%	26.9%	0.834
qwen2-math-1.5b	0.933	0.267	32.3%	37.2%	30.5%	0.903

**Table 4:** Backbones as reported by the inference runtime. All weights are four-bit quantized, and the runtime exposes token log-probabilities but no hidden states.

Backbone	Tag	Parameters	Quantization	Family (Runtime Architecture)
qwen2-math-1.5b	qwen2-math:1.5b	1.5 B	Q4_0	Qwen (qwen2)
gemma4-e2b	gemma4:e2b	5.1 B	Q4_K_M	Gemma 4 (gemma4)
gemma4-e4b	gemma4:e4b	8.0 B	Q4_K_M	Gemma 4 (gemma4)
qwen3.5-9b	qwen3.5:9b	9.7 B	Q4_K_M	Qwen (qwen35)
solar-10.7b	solar:10.7b	11 B	Q4_0	Solar (llama)

**Table 5:** Coverage, selective accuracy, correct yield, and generations per item (Gen./item) of each configuration over all 15 cells. Rows marked *signal only* select the stage-1 greedy answers at the coverage of the gate with k=2. These rows draw no stage-2 samples. The MOME-score row still counts the greedy answer and the probes, which the score needs. The two rows rank these answers by mean token log-probability or by the MOME score $c_{\mathrm{safe}}$ and differ only in this ranking signal. The gate also uses stage-2 samples and majority voting. Its difference from the signal-only rows therefore also includes the effect of the policy. Yield is correct yield. Δ is correct yield minus ungated accuracy. *k* is the number of stage-2 samples.

Configuration	Cov.	Sel. Acc.	Yield	Δ (pp)	Gen./Item
Ungated model	100.0%	69.5%	69.5%	—	1.00
Log-probability, signal only	81.2%	76.4%	62.1%	−7.47	1.00
MOME score, signal only	81.2%	81.9%	66.5%	−3.05	2.97
Stage 1 only (immediate-serve band)	31.5%	93.1%	29.3%	−40.23	2.97
**MOME, *k* = 2**	**81.2%**	**85.8%**	**69.6%**	**+0.11**	**4.34**
**MOME, *k* = 4**	**79.0%**	**88.1%**	**69.6%**	**+0.09**	**5.70**

Bold marks the two settings of the gate reported in this paper (k=2 and k=4).

**Table 6:** Coverage, selective accuracy, correct yield, and generations per item (Gen./item) of the four registered variants at k=4. Three-band designs decline low-scoring items directly. Two-band designs send these items to stage 2. The registered adoption rule rejects any variant whose correct yield is lower than that of the ungated model. Bold marks the design that the rule selected. Table 5 reports this design at k=2 and k=4.

Variant	Cov.	Sel. Acc.	Yield	Gen./Item
Three bands, string voting	69.7%	90.6%	63.1%	4.79
Three bands, equivalence voting	70.4%	90.2%	63.5%	4.79
Two bands, string voting	77.3%	88.5%	68.3%	5.70
**Two bands, equivalence voting **	**79.0%**	**88.1%**	**69.6%**	5.70

**Table 7:** Results of the two-band gate with k=2 for each backbone. Ungated is the accuracy of the ungated model. Yield is correct yield. Δ is correct yield minus ungated accuracy. Gen./item is the mean number of generations per item over the three benchmarks of the backbone.

Backbone	Items	Ungated	Cov.	Sel. Acc.	Yield	Δ (pp)	Gen./Item
gemma4-e2b	900	77.4%	89.6%	87.5%	78.3%	+0.89	4.28
gemma4-e4b	900	83.8%	93.3%	90.1%	84.1%	+0.33	3.95
qwen2-math-1.5b	898	52.2%	65.1%	77.1%	50.2%	−2.00	4.32
qwen3.5-9b	899	86.4%	92.2%	94.2%	86.9%	+0.44	4.60
solar-10.7b	900	47.8%	65.8%	74.0%	48.7%	+0.89	4.54
**All**	**4497**	**69.5%**	**81.2%**	**85.8%**	**69.6%**	**+0.11**	**4.34**

Bold marks the results over all backbones.

**Table 8:** Generations per item for each cell under Equation (1), sorted by cost. The fixed part of the cost is about three generations. The variable part depends only on the share of items served immediately at stage 1. The table shows the two cheapest cells, the three costliest cells, and the mean over all evaluated items.

Cell	Served Immediately	To Stage 2	Gen./Item
GSM8K (en) × gemma4-e4b	64.7%	35.3%	3.69
GSM8K (en) × qwen2-math-1.5b	64.0%	36.0%	3.71
⋯			
MATH-500 (en) × solar-10.7b	10.3%	89.7%	4.72
HRM8K-GSM8K (ko) × qwen3.5-9b	2.3%	97.7%	4.95
HRM8K-GSM8K (ko) × qwen2-math-1.5b	0.7%	99.3%	4.98
**All 15 cells**	**31.5%**	**68.5%**	**4.34**

Bold marks the mean over all 15 cells.

**Table 9:** Coverage, selective accuracy, correct yield and generations per item (Gen./item) for each number *k* of stage-2 samples. The ballot contains the greedy answer and the *k* samples. A tie is possible with an even number of votes. The gate declines an item with a tied vote. Each row uses the first *k* of four fixed sample paths. Bold marks the two settings reported in the paper.

*k*	Votes	Cov.	Sel. Acc.	Yield	Δ (pp)	Gen./Item
0	—	31.5%	93.1%	29.3%	−40.23	2.97
1	2 (even)	71.9%	89.0%	64.0%	−5.58	3.65
**2**	**3 (odd)**	**81.2%**	**85.8%**	**69.6%**	**+0.11**	4.34
3	4 (even)	73.7%	90.1%	66.4%	−3.11	5.02
**4**	**5 (odd)**	**79.0%**	**88.1%**	**69.6%**	**+0.09**	5.70

**Table 10:** MOME score and gate when the fitting data exclude the scored problems, the scored model family, or the scored benchmark. All rows score the same 4497 greedy answers and use the two-band gate with k=2 (vote share ≥0.6). Except in the first row, $\tau_{\mathrm{high}}$ was re-derived with the registered procedure on the fitting data only. ΔAUROC is the item-paired difference to the second row (95% interval, 2000 replicates within cells). S1 prec. is the precision of the answers served at stage 1. Unobs. counts items that a re-fitted score sends to stage 2 but that have no stage-2 samples, because the registered gate served them at stage 1. For these items, the coverage, accuracy, and Δyield columns use the outcome of the 50 observed stage-2 items closest to the serve band (per backbone and greedy label). The last column gives the Δyield if all of them were declined and if all were served with the greedy answer. Δyield is in percentage points against the ungated model.

Fit	AUROC	ΔAUROC	S1 Prec.	Cov.	Sel. Acc.	ΔYield	Unobs.	Range
Backbone-out (registered)	0.863	—	—	81.2%	85.8%	+0.11	0	—
Backbone-out, $\tau_{\mathrm{high}}$ re-derived	0.863	—	93.1%	81.2%	85.8%	+0.11	2	[+0.1,+0.1]
Problem-disjoint, five folds	0.859	[−0.005,−0.002]	92.2%	81.1%	85.8%	+0.02	252	[−5.1,+0.2]
Model family held out	0.863	[−0.002,+0.004]	91.6%	81.7%	85.5%	+0.29	6	[+0.2,+0.3]
Benchmark held out	0.835	[−0.034,−0.022]	86.7%	81.8%	84.7%	−0.30	533	[−11.0,+0.1]

**Table 11:** Four backbones of further families, 900 items each (the same problems as Table 7). The predictor and $\tau_{\mathrm{high}}$ were fitted once on the five backbones of the main evaluation and not changed. Ungated is the accuracy of the greedy answer. The last column is the item-paired difference in AUROC between the MOME score and mean token log-probability, with its 95% interval (2000 replicates within cells). All four backbones are served with four-bit weights (Q4_K_M).

Backbone	Family (Developer)	Parameters	Ungated	AUROC, MOME	AUROC, Mean Log-Prob.	ΔAUROC
llama3.2-3b	Llama 3.2 (Meta)	3.2 B	51.6%	0.823	0.547	+0.276 [+0.240,+0.319]
phi4-mini	Phi-4 (Microsoft)	3.8 B	65.3%	0.852	0.629	+0.223 [+0.183,+0.264]
exaone3.5-7.8b	EXAONE 3.5 (LG AI Research)	7.8 B	81.6%	0.785	0.662	+0.124 [+0.075,+0.171]
granite3.3-8b	Granite 3.3 (IBM)	8.2 B	73.4%	0.782	0.551	+0.231 [+0.187,+0.276]
All four	—	—	68.0%	0.829	0.629	+0.200 [+0.180,+0.219]

**Table 12:** The gate at stage-2 temperature *T* of 0.7 (the setting of the main evaluation) and 0.3, with *k* stage-2 samples. Stage 1 is the same in every row of a block. The five backbones of the main evaluation use their backbone-out thresholds; their samples at 0.3 were drawn for the items that their gate sends to stage 2, with the same prompts and seeds as at 0.7. The four further backbones use the predictor and $\tau_{\mathrm{high}}=0.911$ fitted on the five; per-backbone rows show k=2. Δyield is correct yield minus the ungated accuracy in percentage points, with its 95% interval (2000 replicates within cells).

Backbones	*T*	*k*	Cov.	Sel. Acc.	Δyield	Gen./Item
Main evaluation, five backbones	0.7	2	81.2%	85.8%	+0.11 [−0.53,+0.78]	4.34
Main evaluation, five backbones	0.7	4	79.0%	88.1%	+0.09 [−0.60,+0.82]	5.70
Main evaluation, five backbones	0.3	2	83.1%	84.4%	+0.62 [+0.02,+1.25]	4.34
Main evaluation, five backbones	0.3	4	81.2%	86.4%	+0.60 [−0.07,+1.27]	5.70
Four further families	0.7	2	77.9%	85.0%	−1.81 [−2.72,−0.89]	4.50
Four further families	0.7	4	75.9%	87.6%	−1.42 [−2.36,−0.47]	6.04
Four further families	0.3	2	82.9%	81.7%	−0.19 [−1.00,+0.61]	4.50
Four further families	0.3	4	80.8%	84.1%	−0.03 [−0.92,+0.86]	6.04
llama3.2-3b	0.7	2	58.3%	77.9%	−6.11 [−8.44,−4.00]	4.76
llama3.2-3b	0.3	2	69.4%	74.2%	+0.00 [−2.00,+2.00]	4.76
phi4-mini	0.7	2	76.1%	84.1%	−1.33 [−3.11,+0.67]	4.79
phi4-mini	0.3	2	80.4%	80.0%	−1.00 [−2.78,+0.89]	4.79
exaone3.5-7.8b	0.7	2	92.3%	89.0%	+0.67 [−0.44,+1.78]	3.95
exaone3.5-7.8b	0.3	2	94.3%	86.7%	+0.22 [−0.89,+1.22]	3.95
granite3.3-8b	0.7	2	84.8%	86.1%	−0.44 [−2.33,+1.33]	4.52
granite3.3-8b	0.3	2	87.4%	84.0%	+0.00 [−1.67,+1.56]	4.52

**Table 13:** Contribution of the feature groups to the MOME score. Every row is re-fitted backbone-out exactly as the registered predictor and scores all 4497 greedy answers. *Alone* uses only the group; *without* uses the other 26 features minus the group, with the item-paired 95% interval of the change against all 26 features. Sel. acc. is the accuracy of the answers that the score alone selects at the coverage of the gate with k=2 (81.2%), for the group alone/without the group.

Feature Group	Features	AUROC Alone	AUROC Without	Sel. Acc. Alone/Without
All 26 features	26	0.863	—	81.9%
Invariant probe	4	0.759	0.764 [−0.110,−0.088]	79.7%/77.7%
Must-change probe	10	0.516	0.854 [−0.013,−0.004]	69.4%/81.8%
Both probe groups	14	0.801	0.754 [−0.121,−0.097]	79.7%/77.6%
Probe vs. original text	4	0.729	0.856 [−0.011,−0.003]	76.0%/81.2%
Original-answer statistics	3	0.647	0.844 [−0.026,−0.012]	75.5%/80.6%
Counts and truncation	5	0.405	0.855 [−0.012,−0.003]	71.5%/80.8%

**Table 14:** Rate at which the invariant edit (an appended redundant clause) changed the answer, in English and in Korean. Items are paired across the English and Korean versions of the same 300 problems. Rates are percentages of the paired items in each row. Differences are Korean minus English. The intervals are from an item-paired bootstrap with 2000 replicates.

Backbone	Pairs	English	Korean	Diff. (pp)	95% CI (pp)
qwen2-math-1.5b	298	9.4%	77.5%	+68.1	+62.8, +73.5
solar-10.7b	300	29.7%	43.7%	+14.0	+7.3, +20.3
gemma4-e2b	300	10.0%	23.3%	+13.3	+8.0, +19.0
qwen3.5-9b	299	4.0%	4.3%	+0.3	−2.7, +3.3
gemma4-e4b	300	8.0%	7.3%	−0.7	−4.7, +3.0
**All**	**1497**	**12.2%**	**31.2%**	**+19.0**	**+16.4**, **+21.6**

Bold marks the results over all backbones.

**Table 15:** AUROC for the outcome label of Section 4.12, the correctness of the answer that the gate would deliver, for the MOME score and for a linear probe on hidden states. Column heads give the number of items. In the difference rows, parentheses give 95% item-paired bootstrap intervals (2000 replicates). The linear probe uses the median-AUROC layer, the layer whose AUROC is the median over all layers (layer 0 is the embedding output); the best layer was not selected. Mean token log-probability is computed on the same items with the same outcome label; parentheses in its row give the 95% item-bootstrap interval.

	gemma4-e4b (897)	qwen3.5-9b (899)
Mean token log-probability	0.691 (0.635, 0.750)	0.777 (0.723, 0.827)
Linear probe, median-AUROC layer	0.756 (layer 21 of 0–42)	0.832 (layer 10 of 0–32)
MOME score $c_{\mathrm{safe}}$	0.741	0.855
Combined (rank average)	0.797	0.871
Combined − linear probe	+0.041 (+0.007, +0.076)	+0.040 (+0.012, +0.069)
Combined − MOME score	+0.056 (+0.021, +0.091)	+0.016 (−0.011, +0.043)
Linear probe − MOME score	+0.015 (−0.054, +0.081)	−0.024 (−0.078, +0.028)
Linear probe − mean log-prob.	+0.065 (−0.013, +0.142)	+0.054 (−0.010, +0.116)
Layers where combined − linear probe excludes zero	35 of 43	33 of 33

## Data Availability

The gate implementation, the per-item measurement records and the scripts that regenerate every table and figure in this paper are published at https://github.com/mrbong77-sys/MOME_Public (accessed on 29 October 2026) and archived on Zenodo under https://doi.org/10.5281/zenodo.22854894. This DOI resolves to the current version, and the results reported here are those of release 1.1. The benchmark datasets are publicly available. Prepared item files are not redistributed; the item identifiers and a SHA-256 hash of every edited problem are published, so that a rebuild from the public sources can be checked.

## Supplementary Materials

The following supporting information can be downloaded at: https://github.com/mrbong77-sys/MOME_Public (accessed on 29 October 2026): the full predictor of the MOME score, which consists of the 26 fingerprint features, their fitted coefficients, and the standardization statistics, together with the code and data described in the Data Availability Statement.

## Abbreviations

The following abbreviations are used in this manuscript:

AUROC	area under the receiver operating characteristic curve
CI	confidence interval
MOME	Module of Metamorphic Evidence
p, pp	percentage point
PCA	principal component analysis

## Appendix A. How the Perturbation Engine Edits a Problem

This appendix traces the perturbation engine on the two GSM8K problems of Table 1 and Table 2. The engine works on the problem text only and does not see the model’s answer or the reference answer. It contains no random step, so the same problem text always yields the same edits. It runs in four steps.

1. *Reversal rules.* The rule pack of the problem’s language is applied, and for LaTeX text, the LaTeX pack as well (Section 3.2). A reversal rule is a regular expression with a replacement, such as *twice as* → *half as*. It produces a candidate edit only if its pattern occurs exactly once in the text. Reversal candidates are annotated *high* certainty.
2. *Scaling rules.* Every number written in digits that passes the well-posedness filter of Section 3.2 yields a candidate in which the number is doubled. At most two such candidates are kept, in order of appearance. One number written in words (for example *three* → *six*) can yield a further candidate. Scaling candidates are annotated *medium* certainty.
3. *Invariant edit.* The redundant clause *(All quantities are exactly as stated.)*, or its Korean version, is appended to the text.
4. *Selection.* The must-change edit is the first high-certainty candidate in the order of step 1 or, if there is none, the first medium-certainty candidate in the order of step 2.

*GSM8K problem 6* (Table 1). The English pack finds *twice as* once, so step 1 yields the reversal *twice as* →*half as*. Step 2 yields two scaling candidates, 4→8 and 20→40. Step 4 selects the reversal because it has high certainty, and the correct answer changes from 260 to 140.

*GSM8K problem 0* (caption of Table 2). No reversal pattern occurs. Step 2 yields 16→32 and 2→4 (the price of an egg), and the word candidate is *three*→*six*. Step 4 selects 16→32, the first medium-certainty candidate, and the correct answer changes from 18 to 50. The rule does not claim that the answer doubles (Section 3.2).

The rule that produced each edit, its annotation, and a SHA-256 hash of each edited text are published with the code for every evaluated problem; the engine regenerates the edited texts from the public benchmarks.

## Appendix B

The tables in this appendix give the full values behind statements in Section 3.2, Section 4.1, Section 4.3 and Section 4.7 and the final-state analysis of Section 4.

**Table A1:** Number of problems for which the perturbation engine emits edits after the well-posedness filter, out of 300 problems per benchmark.

Benchmark	Problems	With a High-Certainty Edit	With a Must-Change Edit	No Must-Change Edit
GSM8K (en)	300	59 (20%)	296 (99%)	4
HRM8K-GSM8K (ko)	300	82 (27%)	298 (99%)	2
MATH-500 (en)	300	76 (25%)	277 (92%)	23

**Table A2:** Main outcomes of the two-band gate with k=2 with 95% bootstrap confidence intervals (2000 replicates). Items were resampled *within* each of the 15 cells at the observed cell size.

Quantity	Estimate	95% CI
Coverage	81.2%	[80.3, 82.1]
Selective accuracy	85.8%	[84.7, 86.8]
Correct yield	69.6%	[68.6, 70.7]
Ungated accuracy	69.5%	[68.4, 70.6]
Correct yield − ungated accuracy (pp)	+0.11	[−0.56, +0.76]

**Table A3:** Processing time of the monitor per item, in milliseconds, excluding backbone generation. p50 is the median and p95 the 95th percentile. The total is for the slowest path, in which both probe answers are expressions. Its p95 is the sum of the p95 values of the steps.

Step (per Item)	p50 (ms)	p95 (ms)
Edit generation (perturbation engine)	0.100	0.212
Probe observation, numeric answer	0.001	0.001
Probe observation, expression answer	0.242	2.498
Score and decision (26-feature logistic model)	0.004	0.005
**Monitor total, worst-case path**	**0.59**	**5.21**

Bold marks the total of the worst-case path.

**Table A4:** Final state of every evaluated item and whether the relevant answer was correct. For a served row, the Correct and Wrong columns refer to *the answer that the system returned*. For a declined row, they refer to *the greedy answer that the ungated model would have returned*. This greedy answer is the answer that the decline withheld. Stage 2 changes some answers. The Correct column therefore does not sum to the number of correct answers of the ungated model.

Final State	Items	Correct	Wrong
**Served Rows: The Returned Answer. Declined Rows: The Withheld Greedy Answer.**			
Served at stage 1	1415	1318	97
Served at stage 2	2237	1814	423
Declined, no majority	845	88	757

**Table A5:** Sensitivity of the gate (k=2) to the stage-1 threshold and the stage-2 vote rule (4497 items, registered MOME scores). Upper block: every backbone’s $\tau_{\mathrm{high}}$ shifted by the same amount. A higher threshold sends items to stage 2 that were served at stage 1 in the evaluation and therefore have no stage-2 samples (Unobs.). For these items, the table uses the outcome of the 50 observed stage-2 items closest to the serve band, and the last column gives the Δyield if all were declined or all served with the greedy answer. Lower block: vote rules at the registered thresholds; 3 votes is k=2, 5 votes is k=4. * With five votes, a share of 0.6 requires three agreeing votes. Δyield in percentage points against the ungated model.

Threshold	Vote Rule	Cov.	Sel. Acc.	ΔYield	Gen./Item	Unobs.	Range
$\tau_{\mathrm{high}}-0.10$	3 votes, ≥2 agree	82.6%	84.9%	+0.58	3.73	0	—
$\tau_{\mathrm{high}}-0.05$	3 votes, ≥2 agree	82.1%	85.3%	+0.49	3.90	0	—
$\tau_{\mathrm{high}}-0.02$	3 votes, ≥2 agree	81.7%	85.5%	+0.29	4.10	0	—
$\tau_{\mathrm{high}}+0.00$	3 votes, ≥2 agree	81.2%	85.8%	+0.11	4.34	0	—
$\tau_{\mathrm{high}}+0.01$	3 votes, ≥2 agree	80.9%	85.9%	−0.06	4.47	299	[−6.0,+0.1]
$\tau_{\mathrm{high}}+0.02$	3 votes, ≥2 agree	80.6%	85.9%	−0.26	4.63	645	[−13.1,+0.1]
$\tau_{\mathrm{high}}+0.03$	3 votes, ≥2 agree	80.3%	86.0%	−0.49	4.79	1012	[−20.7,+0.1]
registered	3 votes, plurality	81.9%	85.0%	+0.13	4.34	0	—
registered	3 votes, ≥2 agree	81.2%	85.8%	+0.11	4.34	0	—
registered	3 votes, unanimous	66.0%	92.2%	−8.67	4.34	0	—
registered	5 votes, ≥3 agree *	79.0%	88.1%	+0.09	5.70	0	—
registered	5 votes, ≥4 agree	70.3%	91.9%	−4.94	5.70	0	—
registered	5 votes, unanimous	61.7%	93.3%	−11.94	5.70	0	—
